# Metabolomic signatures after bariatric surgery – a systematic review

**DOI:** 10.1007/s11154-021-09695-5

**Published:** 2021-12-02

**Authors:** Matilde Vaz, Sofia S. Pereira, Mariana P. Monteiro

**Affiliations:** 1 Endocrine & Metabolic Research, Unit for Multidisciplinary Research in Biomedicine (UMIB), University of Porto, Porto, Portugal; 2 Department of Anatomy, School of Medicine and Biomedical Sciences (ICBAS), University of Porto, Porto, Portugal

**Keywords:** Metabolomics, Bariatric surgery, Weight loss, Type 2 diabetes remission

## Abstract

Metabolomics emerged as an important tool to gain insights on how the body responds to therapeutic interventions. Bariatric surgery is the most effective treatment for severe obesity and obesity-related co-morbidities. Our aim was to conduct a systematic review of the available data on metabolomics profiles that characterize patients submitted to different bariatric surgery procedures, which could be useful to predict clinical outcomes including weight loss and type 2 diabetes remission. For that, the Preferred Reporting Items for Systematic Reviews and Meta-Analyses - PRISMA guidelines were followed. Data from forty-seven original study reports addressing metabolomics profiles induced by bariatric surgery that met eligibility criteria were compiled and summarized. Amino acids, lipids, energy-related and gut microbiota-related were the metabolite classes most influenced by bariatric surgery. Among these, higher pre-operative levels of specific lipids including phospholipids, long-chain fatty acids and bile acids were associated with post-operative T2D remission. As conclusion, metabolite profiling could become a useful tool to predict long term response to different bariatric surgery procedures, allowing more personalized interventions and improved healthcare resources allocation.

**Supplementary information:**

The online version contains supplementary material available at 10.1007/s11154-021-09695-5.

## Introduction

The increasing prevalence of obesity in modern society makes it one of the main public health concerns [1]. Obesity is a major risk factor for several other medical conditions and particularly for non-communicable diseases [2–4]. Bariatric surgery is the most effective treatment for severe obesity and its associated co-morbidities, since it has proven to successfully achieve a significant and sustained body mass index (BMI) decrease, besides improving several obesity related diseases, such as type 2 diabetes (T2D) [4]. Although bariatric surgery is a highly effective intervention for obesity treatment in majority of patients, 10–20% of individuals fail to achieve clinically relevant BMI reduction or T2D remission, a percentage that tends to increase along the post-operative timespan [5–7]. In order to prevent the raising number of primary or secondary failures derived from the widespread use of bariatric surgery for obesity and obesity related disorders treatment has rendered the identification of biomarkers able to predict surgery outcomes imperative. This aim became tangible by recent advances in omics technologies, which include genomics, transcriptomics, epigenomics, proteomics, metabolomics, metagenomics, which also provided the opportunity to unravel the mechanisms underlying phenomena, such as weight loss and metabolic improvement. Additionally, these are now well-recognized as crucial research tools to achieve the progress required to enact personalized medicine approaches [8, 9]. Particularly, metabolomics represent a valuable tool to study dynamic metabolic responses and biological systems adaptations to a given stimulus [3, 10]. Metabolomics has been widely used across several scientific areas in the quest for molecular fingerprints that could act as diagnostic and prognostic biomarkers. Metabolomics analyses are usually performed by stand-alone hydrogen nuclear magnetic resonance technique or mass spectrometry technique, combined with different metabolite chromatographic separation methods, such as capillary electrophoresis, liquid or gas chromatography [11]. These metabolite detection methods allow the characterization of low molecular weight metabolites from several different classes [11]. The use of metabolomics in the study of obesity has empowered, not only the understanding of the biochemical and metabolic disruptions underlying this disease condition, but also the acknowledgement of the metabolic and physiological impact of therapeutic interventions, such as bariatric surgery [12]. Metabolomics studies enable to evaluate to what extent does the anatomical modifications of the gastro-intestinal tract induced by bariatric surgery alter the individual’s metabolomics profile [11]. Furthermore, these techniques also allow to understand how different bariatric surgery techniques that result in distinct anatomical rearrangements of the gut, impact on the individuals’ metabolic profiles [13]. Some metabolomics signatures harbor the potential to provide a mechanistic explanation on the heterogeneity of patient outcomes elicited by bariatric surgical interventions [14, 15].

Thus, the aim of this review was to systematize the available data in order to describe the metabolic fingerprints that characterize patients’ submitted to different bariatric surgery procedures. Our goal was also to identify a preoperative or early postoperative metabolite profile potentially useful to predict weight loss response and T2D remission after bariatric surgery.

## Methods

### Protocol and registration

This project was submitted to PROSPERO (registration number CRD42021235341) and it is available at https://www.crd.york.ac.uk/prospero/display_record.php?ID=CRD42021235341. The review was performed according to the Preferred Reporting Items for Systematic Reviews and Meta-Analyses (PRISMA) guidelines [16].

### Information sources and search approach

Publications reporting original data on metabolomics profiles induced by bariatric surgery were searched in three different electronic bibliographic databases: PubMed, Scopus and Isi Web of Knowledge, in February 2021. The detailed search approaches for each database are described in the Supplementary File 1.

### Study selection and inclusion criteria

The main domain of this review were metabolomics studies carried out in patients submitted to bariatric surgery for the primary treatment of obesity and obesity related-disorders. The inclusion criteria comprised studies conducted in adult individuals submitted to bariatric surgery, including Sleeve Gastrectomy (SG), Laparoscopic Adjustable Gastric Band (LAGB), Roux-en-Y Gastric Bypass (RYGB), Duodenal Jejunal Bypass (DJB), Biliopancreatic Diversion (BPD), Duodenal Switch (DS), and Single-anastomosis Duodeno-ileal Bypass with Sleeve Gastrectomy (SADI-S). The exclusion criteria were study data pertaining to children and pregnant women.

### Data extraction

Studies provided by the bibliographic databases were screened to eliminate duplicates. Afterwards, the publications were independently reviewed by two authors, based on title and abstracts, in order to assess the eligibility criteria of each study. Thereafter, full text examination was also required in case of doubt. A third author conducted an independent review of the discordant articles and solved disagreements by majority consensus.

The papers selected for inclusion were scored according to the Newcastle–Ottawa Scale, to assess the quality and risk of bias (Supplementary Files 2 and 3). Only studies considered to be at least of moderate quality, i.e. with a score of 6 or higher, were included for data analysis.

The selected studies were divided among authors for individual data extraction, and later reviewed by other author in a cross-over manner. Data was retrieved and summarized according to the following information: author(s), study name, reference, experimental design, number of subjects enrolled, most relevant patient features (age, sex, BMI and presence of T2D), type of bariatric surgery procedure, type of biological fluid, time points of the sample collection, experimental approach, study outcomes when applicable and major findings.

The articles were also subdivided into four different groups: (1) studies considering preoperative and postoperative metabolomic signatures; (2) studies that disclosure preoperative and early postoperative metabolomic signatures associated with T2D or (3) with weight loss response; (4) studies comparing metabolomic profiles after different bariatric surgery interventions.

## Results

**Fig. 1:**
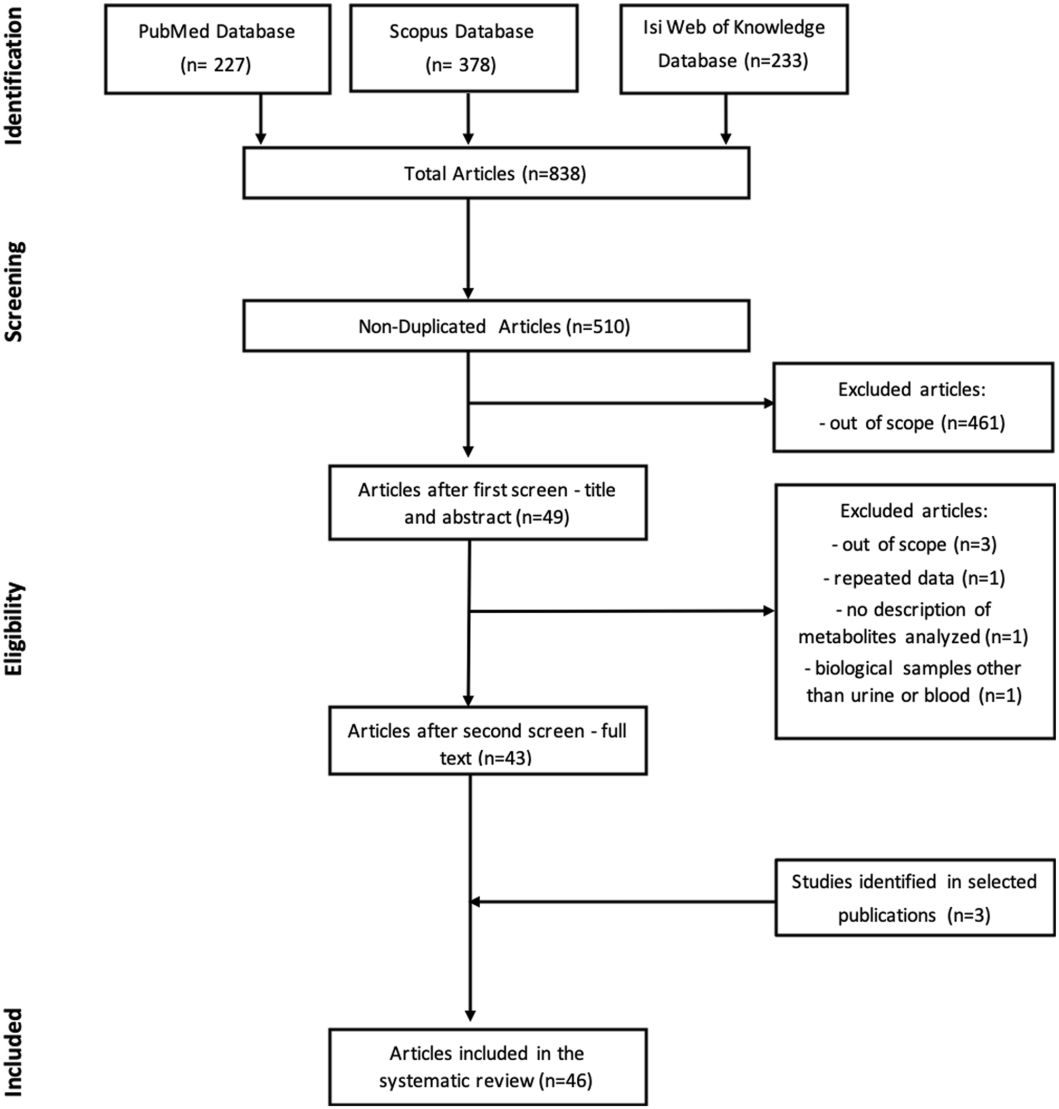
Flowchart of the search, eligibility criteria approaches and study inclusion for systematic review

Our search identified 378 papers in Scopus, 227 in PubMed and 233 in Isi Web of Knowledge, resulting in a total of 838 papers. After elimination of duplicates (*n* = 328), a total of 510 papers were submitted to an initial screen by reading papers’ titles and abstracts by two independent researchers. From those, 461 were out of scope resulting in a total of 50 full-text papers to be evaluated for eligibility. After reading the full texts, 6 papers were eliminated due to the following reasons: repeated data (*n* = 1), out of scope (*n* = 3), no description of metabolites analyzed (*n* = 1) and metabolomics studies conducted in biological fluids other than urine or blood (*n* = 1). Additionally, 3 papers were identified from reading the full texts reference list, resulting in a total of 47 papers to be included in the systematic review (Fig. 1). Among these, 46 studies included conducted metabolomic analysis on plasma or serum and only 1 study was conducted on urine.

### Metabolomic profiles induced by bariatric surgery

**Fig. 2:**
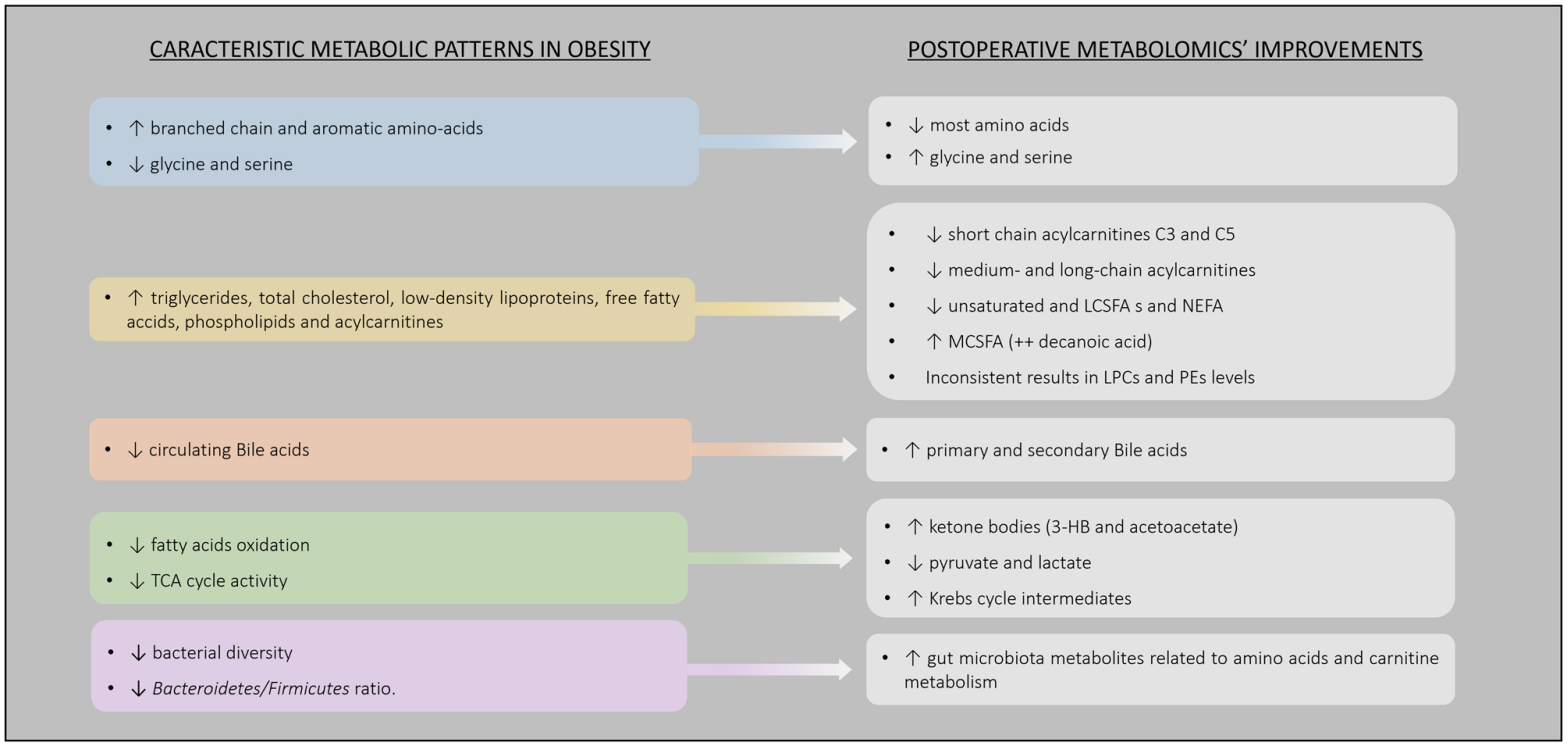
Main metabolomic alterations induced by bariatric surgery, in patients with obesity. Abbreviations: 3-HB – 3-hydroxybutyrate; LCSFA – Long-Chain Saturated Fatty Acids; LPC – Lysophosphatidylcholine; MCSFA – Medium-Chain Saturated Fatty Acids; NEFA – Non-Esterified Fatty Acids; PE – Phosphatidylethanolamine; TCA – Tricarboxylic Acid

Several studies focused on evaluating the changes in metabolomic profile induced by bariatric surgeries (Fig. 2 and Supplementary File 4). Amino acids (AAs), lipids, energy metabolism-related metabolites and gut microbiota-related metabolites were the most frequently studied metabolite classes.

Numerous studies demonstrated that circulating levels of valine [17–34], isoleucine [17–24, 26, 27, 29–36], leucine [17–24, 26, 27, 29–33, 35–37], phenylalanine [17, 18, 20, 21, 23, 24, 26–29, 37], tyrosine [17–21, 23, 24, 27, 29, 30, 37], tryptophan [17, 21, 24, 29, 37, 38], alanine [17–21, 23, 28, 30, 33, 39], proline [17–19, 21, 28, 39], methionine [18, 23, 30], aspartate [21], threonine [19, 21, 32], lysine [19] and ornithine [18, 21, 24, 30, 40] decrease after bariatric surgery. Contrarily, circulating levels of glycine [18–22, 24, 26, 27, 31, 36, 38, 41] and serine [18, 28, 31, 38] were reported to increase after bariatric procedures, while other AAs, such as glutamine, histidine, arginine and asparagine were inconsistently reported as being either increased [20, 24, 27, 36, 41, 42] or decreased [18, 19, 21, 23, 28, 30, 39], after bariatric surgery. No pattern, regarding the type of surgical procedures or the postoperative time, was recognized as potentially responsible for the differences in these specific AA profiles observed across the studies.

Among the lipid class, acylcarnitines (ACs), free fatty acids (FFAs), bile acids (BAs) and phospholipids (PLs) were the metabolites most frequently studied in patients submitted to bariatric surgery interventions.

Acylcarnitines are esters of L-carnitine and fatty acids. The short chain AC acetylcarnitines were reported to increase rapidly after interventions and to remain elevated for 6 to 12 months after bariatric surgery [17, 21, 28, 32, 37, 43]. Contrarily, the short chain acylcarnitines derived from the branched chain amino acids (BCAAs) catabolism, C3 and C5, were shown to decrease after bariatric surgery [17, 18, 28, 30, 33, 34, 43]. A transient increase in long-chain ACs levels was observed two-weeks after surgery [28, 44], while most studies report a decrease in medium- and long-chain ACs over the long-term in patients submitted to bariatric surgery [17, 18, 42, 43].

Analysis of circulating fatty acids’ profiles identified a decrease in unsaturated and long-chain saturated fatty acids and non-esterified fatty acids after bariatric surgery, despite some inconsistent results across different studies [17, 19, 22, 25, 32, 45]. The opposite pattern was observed for medium-chain saturated fatty acids (MCSFA), in particular for the decanoic acid, which was found to increase after surgery [20, 22, 25, 32, 37].

Fasting primary and secondary circulating BA levels were also found to increase significantly after bariatric surgery interventions with a malabsorption component, namely RYGB and BPD [44, 46, 47]. This effect was demonstrated to be even more pronounced in the postprandial for total BA, glycine- and taurine-conjugated BA [48]. In contrast, studies conducted in patients submitted to restrictive procedures yielded inconsistent results, with some studies reporting no differences between pre and postoperative BA levels and others reporting increased levels in fasting and postprandial level of glycine-conjugated BA [47, 49].

Phospholipids are important molecules within the cell membrane structure, which include several subclasses such as sphingomyelins (SMs), lysophosphatidylcholines (LPCs), phosphatidylcholines (PCs) and phosphatidylethanolamines (PEs). SM is the dominant sphingolipid in mammalians cells membranes. After BPD, most saturated and unsaturated SMs were found to be decreased[47]. A decrease in saturated SMs levels [43, 50] and increase in unsaturated SMs levels [37, 43, 50] after RYGB was identified by the majority of studies, although results are not consistent across reports [17, 21, 25, 37, 43–45, 50].

Likewise, an increase in ketone bodies (3-hydroxybutyrate and acetoacetate) levels was also identified after bariatric surgery [19, 27, 37, 38, 43, 51]. Although, carboxylic acids and ketone bodies levels were only observed or were more pronounced in short-term period after surgery [22, 27].

Most studies reported a decrease in pyruvate [27, 28, 35, 41] and lactate [19, 28, 32] levels after bariatric surgery. Contrarily, the Krebs cycle intermediates (citrate [19, 24, 27, 37], succinate [24, 38], fumarate [38, 41], malate [24, 41], oxalacetate [35]) were found to increase after surgery.

Gut microbiota metabolites related to AAs and carnitine metabolism were also found to increase after bariatric surgery. In particular, in the levels of p-cresol [17, 19, 32, 37], indole and indoxyl sulfate [20, 25, 29], trimethylamine N-oxide (TMAO) [20, 27], phenol sulfate [37], 3-indolelactic acid [37] and 4-hydroxy-L-proline [37].

### Metabolomic profiles induced by different bariatric surgeries

Only reports comparing the metabolomic profiles induced by different types of bariatric surgery procedures from parallel arm studies were included in this review section. Nine prospective cohort studies and two cross-sectional studies compared the metabolomic profiles induced by different bariatric procedures [18, 27, 31, 33, 37, 39, 46, 47, 52] or its variants [27, 53] (Table 1). From those, seven studies compared the effects of RYGB with two different restrictive bariatric surgeries: LAGB [18, 31, 50] and SG [27, 33, 37, 39], at several time points after surgery, ranging from 3 days to 1 year. Metabolomic signatures associated with each type of bariatric surgery procedure were found. However, one study reported that metabolic signatures differences between RYGB and SG tend to be less prominent 12 months after surgery [37]. When comparing the two types of surgeries, changes in AAs and gut microbiota-related metabolites were the most differentially altered metabolite classes [27, 31, 33, 37, 39]. In addition, a rapid decrease in the majority of lipid classes was observed after both RYGB and LAGB surgeries in the short-term (1 month). However, some PC and SM species returned or tended to return to baseline values 3 months after LAGB, but not after RYGB [50].

**Table 1:** Summary of parallel arm studies comparing the metabolomic profiles induced by different bariatric surgery interventions

FIRST AUTHOR AND YEAR	EXPERIMENTAL DESIGN	PARTICIPANTS	SURGERY GROUPS	AGE AT SURGERY (YEARS)	FEMALE: MALE	PRE-OPERATIVE BMI (kg/m^2^)	TIME AFTER SURGERY	POST-OPERATIVE BMI (kg/m^2^)	SAMPLE TYPE	METABOLOMIC METHOD	MAIN FINDINGS
**LAGB vs RYGB**											
Magkos et al. (2013) [18]	Prospective cohort study	Patients with obesity and NGT (*N* = 20)	LAGB (*n* = 10)	47 ± 14	9:1	46.5 ± 8.8	22 ± 7 weeks	37.6 ± 7.3	Fasting plasma	Targeted MS/MS (AAs and acylcarnitine)	↓ BCAAs and acylcarnitines after both surgeries in similar proportions
			RYGB (*n* = 10)	43 ± 7	8:2	45.6 ± 6.7	16 ± 2 weeks	36.4 ± 5.0			
Lips et al. (2014) [31]	Prospective cohort study	Patients with obesity and NGT (*N* = 74: *n* = 27 submitted to bariatric surgeries)	LAGB (*n* = 11)	46.3 ± 1.9	11:0	43.1 ± 0.9	3 weeks	41.10 ± 0.85	Fasting plasma	Targeted UPLC–tandem MS (AAs analysis)	↓ BCAAs after both surgeries ↓ BCAAs was higher in patients submitted to RYGB ↑ Glycine and serine after RYGB ↓ AAAs after RYGB
							3 months	39.02 ± 0.82			
			RYGB (*n* = 16)	48.6 ± 1.6	16:0	44.2 ± 0.8	3 weeks	40.48 ± 0.85			
							3 months	36.63 ± 0.82			
Kayser et al. (2017) [50]	Prospective cohort study	Patients with obesity and at least one severe obesity-related comorbidity (*N* = 59)	LAGB (*n* = 22)	34.5 ± 1.6	22:0	43.6 ± 0.7	1 month	40.4 ± 1.0	Fasting serum	Targeted LC–MS/MS (lipidomic)	↓ equivalently in the majority of lipids between both surgical groups ↓ some PC and SM species at 1 month after surgery and remained suppressed 3 months after RYGB, while either returned or tended to return to baseline values 3 months after LAGB
							3 months	38.3 ± 1.0			
			RYGB (*n* = 37)	37.3 ± 1.9	37:0	46.5 ± 1.0	1 month	41.1 ± 1.1			
							3 months	38.0 ± 1.2			
**SG vs RYGB**											
Jüllig et al. (2014) [39]	Prospective cohort study	Patients with obesity and T2D (*N* = 15)	SG (*n* = 7)	46.8 ± 2.9	6:1	42.1 ± 4.0	3 days	NA	Fasting plasma	Untargeted GC–MS	↓ histidine, proline, citrate and decanoic acid after RYGB ↑ 2-hydroxybutyrate and 3-methyl-2-oxo-pentanoic acid after SG
			RYGB (*n* = 8)	41.0 ± 3.1	8:0	42.1 ± 4.0	3 days	NA			
Samczuk et al. (2018) [37]	Prospective cohort study	Patients with obesity and T2D (*N* = 54)	SG (*n* = 34)	49.3 ± 8.7	14:20	50.92 ± 7.33	1 month	45.90 ± 6.9	Fasting serum	Untargeted GC–MS and LC–MS	↓ AAAs induced by SG was higher ↑ P-cresol after SG ↑ sulfate-containing metabolites after RYGB ↓ PC, LPC, PE and LPE after both surgeries ↑ sphingomyelins and choline after both surgeries
							6 months	37.33 ± 7.3			
			RYGB (*n* = 20)	50.1 ± 9.3	15:5	45.79 ± 5.5	1 month	40.97 ± 5.15			
							6 months	32.61 ± 5.5			
Tan et al. (2016) [33]	Prospective cohort study	Patients with obesity^*^ (*N* = 22, 13 with T2D)	SG (*n* = 12)	36.3 ± 8	13:9	38.8 ± 1.3	12 months	NA	Fasting serum	Targeted LC/MS (AAs and acylcarnitine)	↓ BCAAs after both surgeries in similar proportions ↓ AAAs after SG
			RYGB (*n* = 10)	45.6 ± 9.1			12 months	NA			
Gralka et al. (2015) [27]	Prospective cohort study	Patients with obesity (*N* = 106)	SG (*n* = 19)	43.6 ± 1.0	73:33	53.6 ± 10.0	3 months	46.0 ± 10.1	Fasting serum	Untargeted ^1^H-NMR	Metabolomic effect of RYGB are higher compared with SG ↓ BCCAs and AAAs after both bariatric surgeries. ↓ in valine was weaker after SG Carboxylic acids anions levels were modified similarly by the different bariatric surgeries ↑ TMAO after SG ↑ in dimethyl sulfates after surgery was higher in the distal RYGB
							6 months	38.3 ± 10.0			
							9 months	33.3 ± 6.1			
							12 months	31.7 ± 4.1			
			Proximal RYGB (*n* = 27)			43.0 ± 5.8	3 months	36.7 ± 5.5			
							6 months	32.6 ± 5.4			
							9 months	30.0 ± 5.5			
							12 months	29.4 ± 5.9			
			Distal RYGB (*n* = 60)			46.3 ± 6.5	3 months	39.1 ± 5.5			
							6 months	35.2 ± 5.4			
							9 months	32.4 ± 5.2			
							12 months	30.7 ± 4.9			
**RYGB with short BPL vs RYGB with long BPL**											
Jarak et al. (2020) [53]	Cross-seccional study	Patients with obesity and NGT (*N* = 20)	RYGB with short BPL (*n* = 9)	38 ± 3	8:1	41.8 ± 1.1	1.6 ± 0.3 years	28.1 ± 2.3	Fasting and post-prandial plasma	Untargeted ^1^H-NMR	Fasting similar global profiles between groups ↑ post-prandial acetate in patients submitted to RYGB with long BPL
			RYGB with long BPL (*n* = 11)	43 ± 2	10:1	40.6 ± 0.9	1.5 ± 0.3 years	26.2 ± 2.8			
**SG vs BPD**											
Ramos-Molina et al. (2018) [47]	Prospective cohort study	Patients with obesity and NGT (*N* = 37)	SG (*n* = 25)	47.0 ± 6.7	16:9	47.9 ± 6.1	6 months	36.5 ± 4.5	Fasting plasma	Targeted UPLC-MS (lipidomic)	Lipidomic profiles induced by the two surgeries are different BPD: ↓ sphingolipids and phospholipids; ↑ bile acids SG: ↑ sphingolipids and phospholipids; no changes in bile acids levels
			BPD (*n* = 12)	44.4 ± 8.2	6:6	51.8 ± 6.9	6 months	39.7 ± 4.4			
**BPD-DS vs RYGB**											
Ahlin et al. (2019) [46]	Prospective cohort study	Patients with obesity and NGT (*N* = 15)	BPD (*n* = 9)	44.7 ± 8.1	5:4	55.8 ± 9.5	183.7 ± 61.8	39.1 ± 8.5	Fasting plasma	Targeted UPLC-MS (bile acids analysis)	No differences in fasting bile acid levels were observed between the RYGB and BPD groups
			RYGB (*n* = 6)	43. 7 ± 9.4	0:6	45.3 ± 5.7	187.8 ± 93.6	35.5 ± 5.5			
**BPD-DS vs SADI-S**											
Pereira et al. (2020) [52]	Cross-seccional study	Patients with obesity and NGT (*N* = 18)	BPD-DS (*n* = 9)	36 ± 12	6:3	51.9 ± 4.0	1.6 ± 0.3 years	29.7 ± 4.3	Fasting and post-prandial plasma	Untargeted ^1^H-NMR	Fasting similar global profiles between groups ↑ post-prandial BCAAs in SADI-S group
			SADI-S (*n* = 9)	43 ± 7	7:2	52.0 ± 3.7	1.5 ± 0.3 years	30.0 ± 3.6			

*AA* amino acids, *AAA* aromatic amino acids, *BCCA* branched chain amino acids, *BMI* body mass index, *BPD* biliopancreatic diversion, *BPD-DS* biliopancreatic diversion with duodenal switch, *BPL* biliopancreatic limb, *GC* gas chromatography, ^*1*^*H-NMR* proton nuclear magnetic resonance, *LAGB* laparoscopic adjustable gastric band, *LC* liquid chromatography, *MS* mass spectrometry, *NA* not available, *NGT* normal glucose tolerance, *PC* phosphatidylcholines, *LPC* lysophosphatidylcholines, *PE* phosphatidylethanolamines, *SG* sleeve gastrectomy, *RYGB* roux-en-Y gastric bypass, *SADI-S* single anastomosis with duodeno-Ileal bypass with sleeve gastrectomy, *T2D* type 2 diabetes, *TMAO* trimethylamine N-oxide, *UPLC* ultra-performance liquid chromatography

^*^Asian cohort

One study that compared the effect of restrictive (SG) and malabsorptive (BPD) bariatric surgeries on metabolomics found that sphingolipids, PLs and BAs levels were differentially altered by the two bariatric surgery procedures. BPD induced an overall decline in sphingolipids and PLs and an increase in BAs levels. Contrarily, SG induced an increase in sphingolipids and PLs and no changes in BAs levels [47].

In contrast, in another study comparing RYGB and BPD, BAs levels were found to be similar after both surgeries [46].

Comparison of fasting and postprandial metabolomics profile of patients submitted to two different malabsorptive surgeries (SADI-S and BPD-DS) was explored in a cross-sectional study. Higher postprandial BCAAs levels after SADI-S, the least malabsorptive surgery, was the only difference observed [52].

The effects of two different RYGB variants on circulating metabolomics profiles was evaluated in a cross-sectional study and in another prospective study. However, the differences in RYGB limb lengths used in the two studies do not allow direct comparisons [53]. Gralka et al. included patients submitted either to a proximal RYGB consisting of a biliopancreatic limb (BPL) of 60 cm and an alimentary limb of 150 cm; or to a distal RYGB that had both BPL and common limb lengths of 60–100 cm [27, 53]. In contrast, the RYGB variants described by Jarak et al. had different BPL length (short-BPL: 60–100 cm vs long-BPL: 200 cm) but the same alimentary limb length (120 cm) [53]. Despite the dissimilarities between RYGB variants, no differences on the postoperative AAs levels were noticed. Both studies found diverse changes in gut microbiota-related metabolites, namely acetate and dimethyl sulfone, when comparing the RYGB variants [27, 53].

### Metabolomic signatures associated with post-bariatric weight loss response

**Fig. 3:**
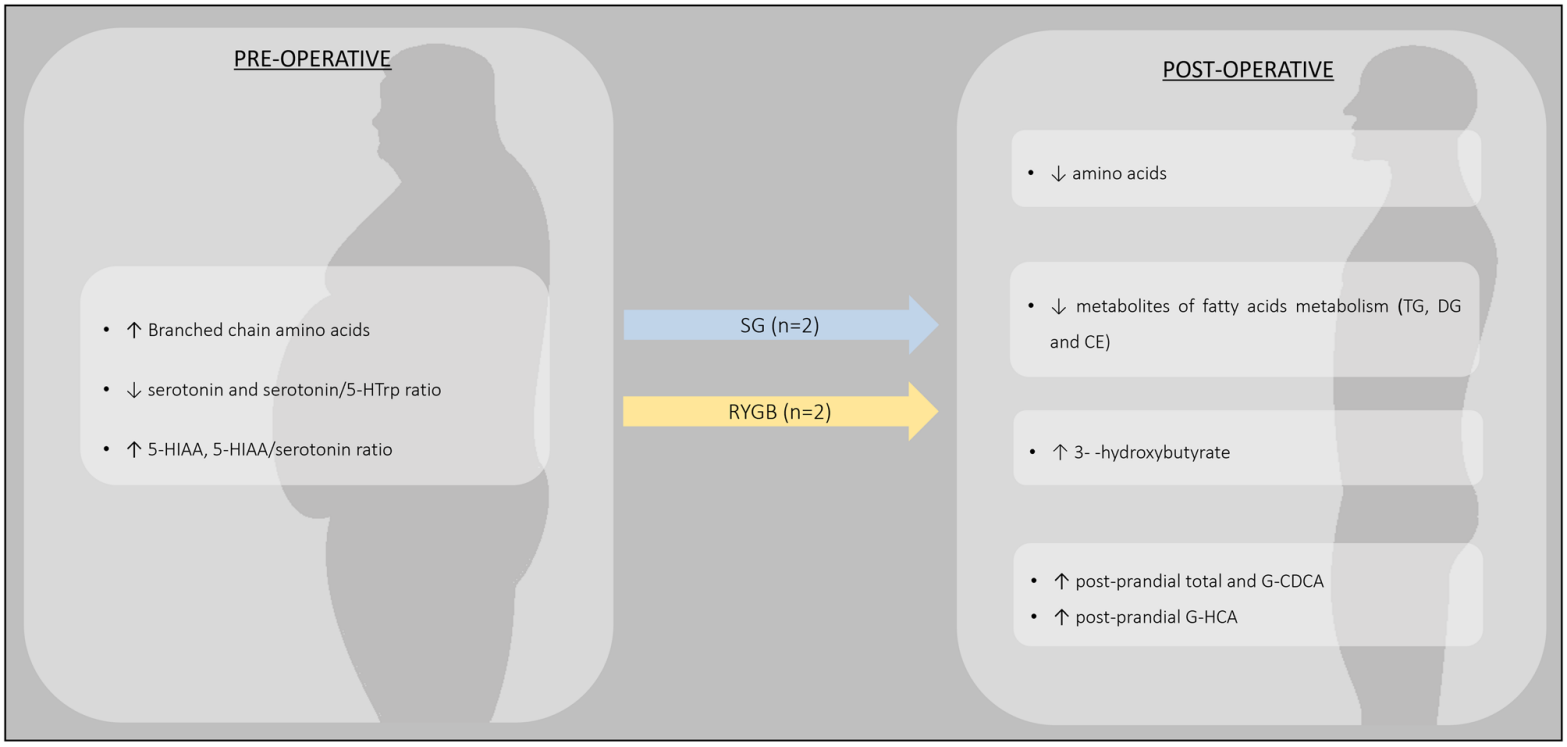
Metabolomic profiles of patients with successful weight loss/maintenance. Abbreviations: 5-HIAA – 5-hydroxyindoleacetic acid; 5-HTrp – 5-hydroxytryptophan; CDCA – Chenodeoxycholic Acid; CE – cholesterol esters; DG – diaglycerols; G- – glycine amidated; HCA – hyocholic acid, SG – Sleeve gastrectomy; RYGB – Roux-en-Y Gastric Bypass; TG – triacylglyceride

The analysis of pre and postoperative metabolomics according to post-bariatric weight loss responses was assessed in 4 independent prospective studies, conducted in patients submitted to SG [49, 54] or RYGB [20, 55] (Fig. 3 and Supplementary File 5).

AAs were the metabolic class that portrait more pronounced changes after bariatric surgeries [20, 54, 55]. Higher baseline BCAAs levels, in particular isoleucine, were correlated with greater weight loss at 3 and 6 months after SG [54]. In the postoperative period, there was a significant decrease in creatine, ornithine, arginine and valine levels in the sub-group of patients with greater weight loss 1 year after RYGB [20].

Moreover, Abidi et al. compared metabolite pattern of patients with sustained weight loss with those of patients who experienced weight regain years after RYGB. The study unraveled that metabolomics’ profile of patients with poor long-term weight loss outcomes were characterized by lower levels of metabolites related to serine, glycine and threonine pathways; lower metabolites from phenylalanine, alanine and glutamate metabolism; lower levels of TCA cycle’ byproducts, as well as higher levels of AAs. In the opposite analysis, higher glycine levels were found in patients with sustained weight loss [55].

Serotonin, a molecule derived from the AA tryptophan, and its metabolites were also proposed as weight loss response molecular fingerprints. A preoperative profile with lower serotonin levels and serotonin/5-hydroxytryptophan (5-HTrp) ratio, and higher 5-hydroxyindoleacetic acid (5-HIAA) levels and 5-HIAA/serotonin ratio were identified in patients with greater weight loss at 3 and 6 months after SG [54].

Patients with sustained weight loss also presented upregulated lipolysis and consequently depletion of triacylglycerols, diaglycerols and cholesterol esters, in addition to higher levels of 3-hydroxybutyrate [55].

Increase in multiple BAs subtypes, more pronounced in postprandial rather than fasting period, was found to correlate with early (6 weeks) and short-term (12 weeks) weight loss response after SG. The authors highlighted the augmented postprandial total and G-CDCA, as being significantly correlated to the 6 weeks BMI loss, as well as the increased postprandial G-hyocholic acid, which was significantly correlated to a greater weight loss percentage at both 6 and 12 weeks [49].

### Metabolomic signatures associated with post-bariatric T2D remission

**Fig. 4:**
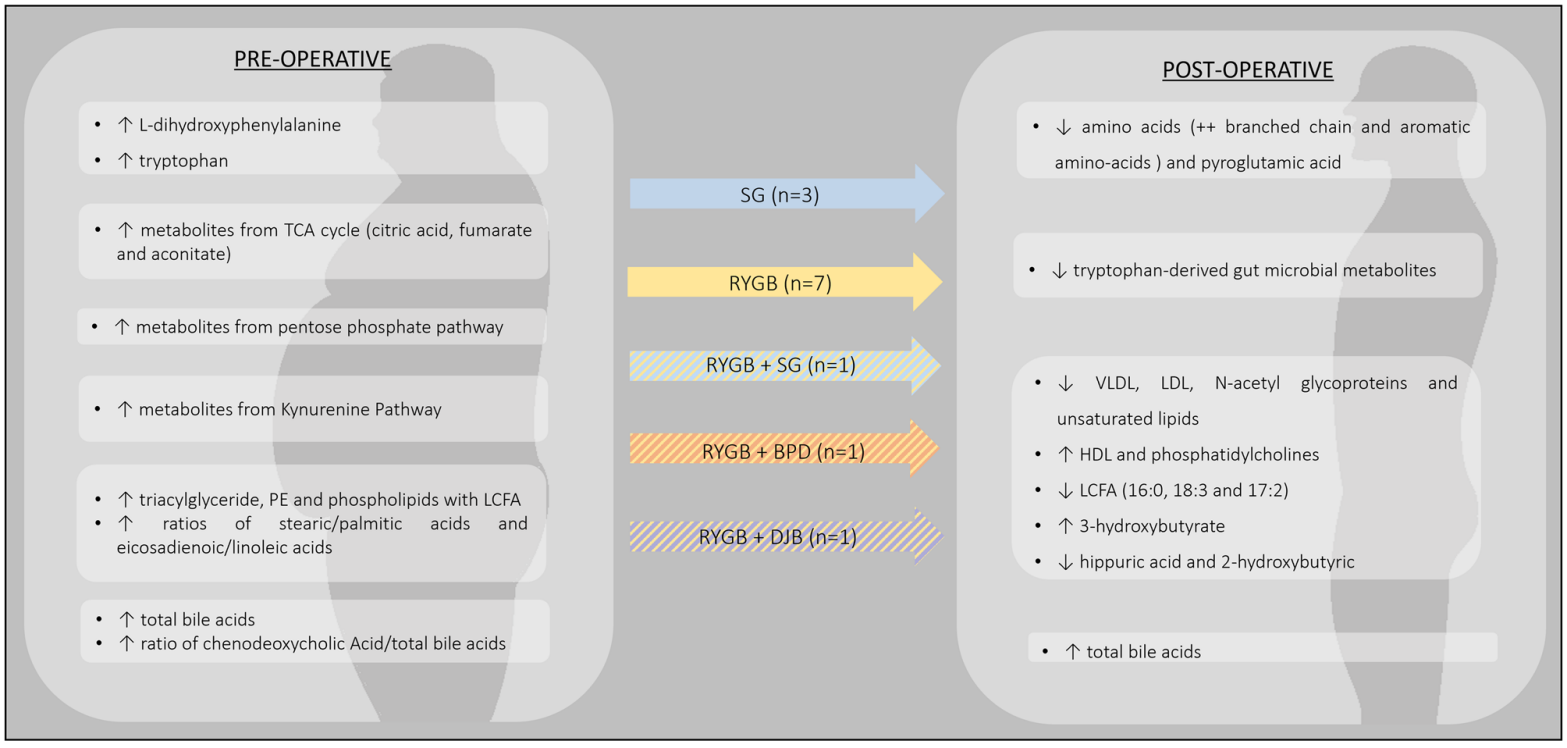
Metabolomic profiles of patients with T2D remission/improved insulin parameters. Abbreviations: BPD – Biliopancreatic diversion; DJB – Duodenal-jejunal bypass; HDL – High-density Lipoprotein; LCFA – long-chain free fatty acids; LDL – Low-density Lipoprotein; PE – Phosphatidylethanolamines; SG – Sleeve gastrectomy; RYGB – Roux-en-Y Gastric Bypass; TCA – Tricarboxylic Acid; VLDL – Very-low-density Lipoprotein

Metabolomics profiles associated with glucose and insulin homeostasis after bariatric surgery were analyzed in fourteen independent prospective cohort studies. While seven papers focused on T2D remission after bariatric surgery, another seven studies addressed the specific profile associated with T2D related parameters, namely glycated hemoglobin (HbA1c) levels, insulin secretion and sensitivity, as inferred from insulinogenic index, homeostasis model assessment-insulin resistance (HOMA-IR) [56] and Quantitative Insulin Sensitivity Check Index (QUICKI) [29]. The majority of studies included individuals submitted either to SG [29, 34, 57] or RYGB [17, 20, 25, 56, 58–61] (Fig. 4 and Supplementary File 5).

Circulating AAs were the most differentially altered metabolite class distinguishing patients that achieved T2D remission from those with persistent disease after bariatric surgical interventions [29, 34, 57, 58, 61, 62]. BCAAs decrease after SG and RYGB, being more pronounced in patients achieving T2D remission [29, 33, 34, 56, 58, 61]. Kwon et al. described, not only an early decline in BCAAS, but also aromatic amino acids (AAAs) [29] in patients depicting improved insulin resistance 3 months after SG.

Preoperative lipid metabolite fingerprints of patients who achieve T2D remission as compared to those without disease remission was characterized by higher PEs, triglycerides and PLs with long-chain fatty acids [25]; while postoperative metabolite profile was characterized by lower very-low-density lipoproteins (VLDL), low-density lipoproteins (LDL), N-acetyl glycoproteins and unsaturated lipids, and increased high-density lipoproteins (HDL) and PCs [17, 57, 58, 63] Arora et al. studied the metabolic profile of patients submitted to RYGB who experienced T2D remission 2 years after surgery [25]. Postoperative fasting plasma lipid profile was characterized by a significant reduction of most lipid species after 4 days and an increase of the same metabolites at 42 days after surgery.

The utility of BAs levels as biomarkers of T2D improvement after bariatric surgery was explored in three independent studies [17, 46, 59]. Two studies addressing T2D remission after RYGB identified higher total BA levels at baseline in the sub-groups of patients with better T2D outcomes [17, 59]. Yu et al. even highlighted that chenodeoxycholic acid (CDCA) and the ratio of CDCA/total BA correlated with T2D remission 2 years after RYGB. Ahlin et al. found that increased total BA levels at 185.3 (± 72.9) days after RYGB and BPD were correlated to insulin resistance improvement regardless the type of surgical procedure [46].

## Discussion

High levels of circulating AAs is a well-known feature of patients with obesity [64]. BCAAs levels are particularly high and experience a rapid decrease after bariatric surgery [17–32, 35–37, 65]. The reduction in BCAA has been attributed to a combination of multiple factors including decreased protein intake; decreased amino acids absorption; increased BCAA catabolism and decreased protein catabolism as a consequence of insulin sensitivity improvement and metabolic amelioration [3]. The upregulation of BCAAs catabolism is supported by the consistent postoperative decline of specific subproducts of BCAAs’ mitochondrial oxidation, namely short chain acylcarnitine’s C3 and C5 [17, 18, 28, 30, 43]. Although BCAAs decrease after both restrictive and malabsorptive surgeries [17–32, 35–37], parallel arm studies reported a greater effect of RYGB in BCAAs levels [27, 31]. Since the differences found in BCAA levels when comparing RYGB and the restrictive procedures seem to be independent of the weight loss, these were hypothesized to be related to impaired AAs absorption induced by the RYGB intestinal rearrangement [27, 31].

Similarly, AAAs were also elevated in patients with obesity and decrease after bariatric surgery [27, 37]. This is consistent with the increase of AAAs derived gut microbiota metabolites (p-cresol, indoxyl sulfate, phenol sulfate and 3-indolelactic acid) after bariatric surgery [17, 19, 20, 29, 32, 37].

Multiple studies reported that BCAAs and AAAs modifications induced by the bariatric procedures could play a relevant role predicting T2D remission [29, 34, 57, 58, 62]. Obesity associated hyperaminoacidemia is a consequence of insulin resistance, which positively affects protein synthesis and proteolysis [66]. BCAAs can also modulate insulin secretion and promote diabetes via hyperinsulinemia, considering its role as insulin secretagogues. Chronic hyperinsulinemia can further stimulate compensatory insulin resistance and potentially lead to pancreatic β-cell exhaustion [67, 68]. Contrariwise, improved insulin sensitivity and secretion after bariatric surgery are associated with decreased circulating AAs [26, 29, 58].

In opposition to most of AAs, the levels of the non-essential AA glycine and serine are known to be low in subjects with obesity and increase after bariatric surgery [18–22, 24, 26–28, 31, 36, 38, 41]. In healthy subjects, glycine is inter-convertible with serine. Glycine participates in multiple biological functions, such as the glutathione synthesis, purines and primary bile salts [69]. Previous studies described low fasting levels of glycine in individuals with impaired glucose tolerance and proposed glycine as an early marker for insulin resistance [70].

Gut microbiota plays an important role in various physiological processes, including metabolism of dietary components and some host-generated substances, with an impact on the use and storage of energy [71]. Gut microbiota of individuals with obesity is characterized by a reduced bacterial diversity and lower ratios of Bacteroidetes to Firmicutes [72]. After bariatric surgery, different gut microbiome-related metabolites arise that differ depending on the type of bariatric procedure. In particular, p-cresol, a metabolite of phenylalanine and tyrosine fermentation by gut microbiota [73, 74], was reported to increase after SG [37]. *Bacteroides fragilis* is one of the bacteria responsible for phenylalanine and tyrosine fermentation. *Bacteroides* were previously demonstrated to increase after SG in individuals with prior T2D diagnosis [75]. So, the effect in tyrosine and phenylalanine levels found in patients with T2D after SG when compared to RYGB, may be related to increased fermentation by gut microbiota. Sulfate-containing metabolites and TMAO, two other gut microbiome-related metabolites were identified to increase after RYGB but not after SG [37]. The differences observed in sulfate-containing metabolites’ results could easily be justified by the fact that the major group of sulfate-reducing bacteria is mainly present in the duodenum, which is trespassed in RYGB [76]. Contrary, the effects of RYGB in TMAO levels are not well understood and unexpected because high TMAO levels have been linked to cardiovascular diseases, whereas RYGB is known to reduce the cardiovascular risk and events [77, 78]. The increase in TMAO levels observed after RYGB, but not after SG, could be explained by the shortening of the small bowel and a less anaerobic metabolism by the gut microbiota, in result of the increase in microbes, such as E.coli and Pseudomonas, responsible for raising TMAO levels [79]. Gut microbiota-related metabolites were also differentially altered by RYGBs with different absorptive limbs. Gralka et al. found that RYGB with a shorter common limb led to a higher increase in dimethyl sulfone levels compared to a more absorptive RYGB [27]. A different study also found that postprandial levels of acetate were higher in patients submitted to RYGB with a longer BPL [53]. Both metabolites are produced in the colon by bacterial fermentation [80], which is enhanced by the higher quantity of undigested food that reaches the colon in the less absorptive RYGB.

The obesity-associated energy metabolic disruption is characterized by decreased oxidation of ketone bodies and down-regulation of TCA cycle [81]. Metabolomic analysis suggests that after bariatric surgery a strong and early upregulation of catabolism and lipolytic activity occurs. Most of intermediate and end products of β-oxidation and ketogenesis increase after intervention, in particular in short-term period after surgery [19, 27, 28, 37, 38, 43, 44, 51]. These findings support a shift in energy metabolism from anabolic to catabolic status, possibly triggered by surgery and caloric restriction.

Obesity is also characterized by a blunted postprandial BAs response. Bariatric surgery, in particular RYGB and BPD normalizes that response, leading to an increase in postprandial BAs that can be justified by the accelerated delivery to the distal intestine where BAs are absorbed [46, 48, 59]. Since BPD leads to an earlier delivery of food to the more distal gut, BPD would be expected to have a higher effect on BAs levels when compared to RYGB. However, BAs levels were found to be similar after both bariatric surgeries [46]. Accordingly, bile diversions to the mid-jejunum or mid-ileum in rats led to the same BAs plasma levels, suggesting that BAs absorption is not necessarily proportional to the length of the gut segment [82]. BAs are reported to have a key role on mediating the anti-diabetic effects of the bariatric surgery. Indeed, higher baseline levels of CDCA were correlated with better outcomes after RYGB [17, 59]. In addition, higher postoperative values of conjugated secondary BAs were associated with an increase of insulin sensitivity after both RYGB and BPD [46]. BAs metabolic effects are mainly mediated by the activation of two receptors: Farnesoid X Receptor (FXR) and Takeda G protein-coupled receptor 5 (TGR5). TGR5 activation in the enteroendocrine cells increases the release of the incretin hormone, glucagon-like peptide-1, known for its anti-diabetic effects [83]. In addition, the receptor TGR5 is also expressed in human muscle and brown adipose tissue, leading to an increase of muscle energy expenditure via T4-T3 conversion, which can further contribute to increase insulin sensitivity [84, 85].

Circulating levels of fatty acids, acylcarnitines, phospholipids, triglycerides, total cholesterol, and LDL are frequently elevated in individuals with obesity and particularly with obesity-related metabolic disorders [64]. Bariatric surgery induces an overall improvement in lipid profile with a marked decrease in most lipid classes. Apart from this trend stands the postoperative increase of MCSFA, known for suppressing fat deposition due to enhanced thermogenesis and fat oxidation in animals and humans [86]. Additionally, previous studies proposed the pharmacological potential of MCSFA for preserving insulin sensitivity in T2D [87, 88].

A preoperative lipid profile with higher levels of PEs, triglycerides and PLs with long-chain fatty acid could also be useful to assess the likelihood of T2D remission after surgery. Long-chain fatty acid metabolism is known to be abnormal in diabetes, leading to impaired formation of specific fatty acids dependent on delta-9, delta-6, and delta-5 desaturation, as well as on chain elongation [89]. Thereby, greater levels of triglycerides with higher carbon number are associated with a decreased risk of T2D [90]. Palmitic acid (FFA 16:0) is also strongly associated with T2D, since this main even-chain fatty acid is significantly decreased before [17] and after [60] RYGB in patients with better T2D outcomes. Palmitic acid was shown to activate Toll-Like Receptor 4 (TLR4) and, subsequently prompt inflammatory cytokines and lipotoxicity in pancreatic β cells, with consequent impaired insulin action [91].

Lipidomic profiles induced by BPD and SG were reported to be distinct [47]. Most sphingolipids and PLs decrease after BPD, which can be attributed to decreased lipid absorption after surgery. On the other hand, PLs levels increased after SG. Circulating levels of PLs are reduced in patients with obesity, which is associated with an increased oxidative status [92, 93]. SG seems to efficiently restore PLs levels to those found in subjects with normal weight by decreasing patients’ oxidative profile. Sphingolipids levels also increase after SG. However, this observation is more difficult to understand since sphingolipids are associated to obesity-related disorders such as insulin resistance and cardiovascular diseases [94].

Nonetheless, the interpretation of the summarized data must be evaluated in the context of the limitations of the reported studies. There are some discrepant results across different analysis, raising the possibility of some bias, independent from the anatomical rearrangements of the surgery itself, such as patients’ dietary habits, which could also have a significant impact on the metabolome. Additionally, it is important to mention that the time points of assessment before and after surgical interventions differed substantially across the studies, in some cases, the follow-up was not long enough for an adequate appreciation of patients’ outcomes. Most of the studies reported the metabolomic profile induced by a single bariatric surgery procedure. Although comparison between studies can be made for understanding the metabolomic profile induced by different surgeries, it raises several constraints derived from the comparison of non-paired patients regarding pre and postoperative characteristics and from the use of different methodological approaches.

In addition, there is still a small number of studies analyzing the importance of metabolomics as predictors of bariatric surgery outcomes. Furthermore, most studies include a small number of patients with short post-operative periods and some of the conclusions are based on the analysis of patients submitted to different technical procedures.

In order to identify metabolomic signatures to predict bariatric surgery response, prospective studies with larger cohorts and longer follow-up periods are needed. Ideally, studies should avoid the inclusion of subjects with heterogenous pre-operative characteristics and submitted to different bariatric surgery. Food intake dairies prior to the analysis should also be taken into account as an additional source of bias.

By conducting these research trials different patient subgroups metabolomics profiles could be compared and correlated with surgical outcomes, thus harboring the potential to identify a pre-operative metabolomic pattern that could act as a decision aid for the surgeon to choose the bariatric surgery procedure that is more likely to achieve successful weight loss and T2D improvement.

## Conclusions

This review summarizes the impact of bariatric surgery on the individuals’ metabolomics profile and how different bariatric procedures result in distinct metabolomics signatures, which could contribute to explain the heterogeneity of surgical outcomes. Some preoperative metabolomics fingerprints were identified as harboring the potential to be used as prognostic biomarkers for weight loss response and T2D remission. Among these, higher pre-operative levels of lipids including phospholipids, long-chain fatty acids and bile acids are associated with post-operative T2D remission.

## Supplementary information

Below is the link to the electronic supplementary material.

Supplementary file1 (PDF 159 KB)

Supplementary file2 (PDF 197 KB)

Supplementary file3 (PDF 131 KB)

Supplementary file4 (PDF 234 KB)

Supplementary file5 (PDF 216 KB)

## Data Availability

All data is included in the manuscript.

## Abbreviations

BMI: Body mass index
T2D: Type 2 diabetes
PRISMA: Preferred reporting items for systematic reviews and meta-analyses
SG: Sleeve gastrectomy
LAGB: Laparoscopic adjustable gastric band
RYGB: Roux-en-Y gastric bypass
DJB: Duodenal jejunal bypass
BPD: Biliopancreatic diversion
DS: Duodenal switch
SADI-S: Single-anastomosis duodeno-ileal bypass with sleeve gastrectomy
AA: Amino acid
AC: Acylcarnitine
FFA: Free fatty acid
BA: Bile acid
PL: Phospholipid
BCAA: Branched chain amino acid
MCSFA: Medium-chain saturated fatty acid
SM: Sphingomyelins
LPC: Lysophosphatidylcholine
PC: Phosphatidylcholine
PE: Phosphatidylethanolamine
TMAO: Trimethylamine N-oxide
HbA1c: Glycated hemoglobin
HOMA-IR: Homeostasis model assessment-insulin resistance
QUICKI: Quantitative insulin sensitivity check index
AAA: Aromatic amino acid
3-HAA: 3-Hydroxyanthranilic acid
L-DOPA: L-dihydroxyphenylalanine
TCA: Tricarboxylic acid
VLDL: Very-low-density lipoprotein
LDL: Low-density lipoprotein
HDL: High-density lipoprotein
CDCA: Chenodeoxycholic acid
5-HTrp: 5-Hydroxytryptophan
5-HIAA: 5-Hydroxyindoleacetic acid
BPL: Biliopancreatic limb
FXR: Farnesoid X receptor
TGR5: Takeda G protein-coupled receptor 5
TLR4: Toll-like receptor 4

## References

[1] Rangel-Huerta OD, Pastor-Villaescusa B, Gil A (2019). Are we close to defining a metabolomic signature of human obesity? A systematic review of metabolomics studies. Metabolomics.

[2] Nyberg ST, Batty GD, Pentti J, Virtanen M, Alfredsson L, Fransson EI, Goldberg M, Heikkilä K, Jokela M, Knutsson A, Koskenvuo M, Lallukka T, Leineweber C, Lindbohm JV, Madsen IEH, Magnusson Hanson LL, Nordin M, Oksanen T, Pietiläinen O, Rahkonen O, Rugulies R, Shipley MJ, Stenholm S, Suominen S, Theorell T, Vahtera J, Westerholm PJM, Westerlund H, Zins M, Hamer M, Singh-Manoux A, Bell JA, Ferrie JE, Kivimäki M (2018). Obesity and loss of disease-free years owing to major non-communicable diseases: a multicohort study. The Lancet Public Health.

[3] Tulipani S, Griffin J, Palau-Rodriguez M, Mora-Cubillos X, Bernal-Lopez RM, Tinahones FJ, Corkey BE, Andres-Lacueva C (2016). Metabolomics-guided insights on bariatric surgery versus behavioral interventions for weight loss. Obesity (Silver Spring).

[4] Poirier P, Cornier MA, Mazzone T, Stiles S, Cummings S, Klein S, McCullough PA, Ren Fielding C, Franklin BA (2011). Bariatric surgery and cardiovascular risk factors: a scientific statement from the American Heart Association. Circulation.

[5] Arterburn DE, Bogart A, Sherwood NE, Sidney S, Coleman KJ, Haneuse S, O'Connor PJ, Theis MK, Campos GM, McCulloch D, Selby J (2013). A multisite study of long-term remission and relapse of type 2 diabetes mellitus following gastric bypass. Obes Surg.

[6] Courcoulas AP, King WC, Belle SH, Berk P, Flum DR, Garcia L, Gourash W, Horlick M, Mitchell JE, Pomp A, Pories WJ, Purnell JQ, Singh A, Spaniolas K, Thirlby R, Wolfe BM, Yanovski SZ (2018). Seven-year weight trajectories and health outcomes in the longitudinal assessment of bariatric surgery (LABS) study. JAMA Surg.

[7] Christou NV, Look D, Maclean LD (2006). Weight gain after short- and long-limb gastric bypass in patients followed for longer than 10 years. Ann Surg.

[8] Sun YV, Hu YJ (2016). Integrative analysis of multi-omics data for discovery and functional studies of complex human diseases. Adv Genet.

[9] Olivier M, Asmis R, Hawkins GA, Howard TD, Cox LA (2019). The need for multi-omics biomarker signatures in precision medicine. Int J Mol Sci.

[10] Jacob M, Lopata AL, Dasouki M, Abdel Rahman AM (2019). Metabolomics toward personalized medicine. Mass Spectrom Rev.

[11] Samczuk P, Ciborowski M, Kretowski A (2018). Application of metabolomics to study effects of bariatric surgery. J Diabetes Res.

[12] Luo JN, Sheu EG (2020). Do serum metabolites predict weight regain following bariatric surgery?. Dig Dis Sci.

[13] Peterli R, Steinert RE, Woelnerhanssen B, Peters T, Christoffel-Courtin C, Gass M, Kern B, von Fluee M, Beglinger C (2012). Metabolic and hormonal changes after laparoscopic Roux-en-Y gastric bypass and sleeve gastrectomy: a randomized, prospective trial. Obes Surg.

[14] Floegel A, Stefan N, Yu Z, Mühlenbruch K, Drogan D, Joost HG, Fritsche A, Häring HU, de Angelis MH, Peters A, Roden M, Prehn C, Wang-Sattler R, Illig T, Schulze MB, Adamski J, Boeing H, Pischon T. Identification of serum metabolites associated with risk of type 2 diabetes using a targeted metabolomic approach. Diabetes. 2013;62(2):639–48. 10.2337/db12-0495. 10.2337/db12-0495 PMC3554384 23043162

[15] Malin SK, Kashyap SR (2016). Effects of various gastrointestinal procedures on β-cell function in obesity and type 2 diabetes. Surg Obes Relat Dis.

[16] Page MJ, McKenzie JE, Bossuyt PM, Boutron I, Hoffmann TC, Mulrow CD, Shamseer L, Tetzlaff JM, Akl EA, Brennan SE, Chou R, Glanville J, Grimshaw JM, Hróbjartsson A, Lalu MM, Li T, Loder EW, Mayo-Wilson E, McDonald S, McGuinness LA, Stewart LA, Thomas J, Tricco AC, Welch VA, Whiting P, Moher D (2021). The PRISMA 2020 statement: an updated guideline for reporting systematic reviews. PLoS Med.

[17] Luo P, Yu H, Zhao X, Bao Y, Hong CS, Zhang P, Tu Y, Yin P, Gao P, Wei L, Zhuang Z, Jia W, Xu G (2016). Metabolomics study of Roux-en-Y gastric bypass surgery (RYGB) to treat type 2 diabetes patients based on ultraperformance liquid chromatography-mass spectrometry. J Proteome Res.

[18] Magkos F, Bradley D, Schweitzer GG, Finck BN, Eagon JC, Ilkayeva O, Newgard CB, Klein S (2013). Effect of Roux-en-Y gastric bypass and laparoscopic adjustable gastric banding on branched-chain amino acid metabolism. Diabetes.

[19] Mutch DM, Fuhrmann JC, Rein D, Wiemer JC, Bouillot JL, Poitou C, Clément K (2009). Metabolite profiling identifies candidate markers reflecting the clinical adaptations associated with Roux-en-Y gastric bypass surgery. PLoS One.

[20] Narath SH, Mautner SI, Svehlikova E, Schultes B, Pieber TR, Sinner FM, Gander E, Libiseller G, Schimek MG, Sourij H, Magnes C (2016). An untargeted metabolomics approach to characterize short-term and long-term metabolic changes after bariatric surgery. PLoS One.

[21] Palau-Rodriguez M, Tulipani S, Marco-Ramell A, Miñarro A, Jáuregui O, Sanchez-Pla A, Ramos-Molina B, Tinahones FJ, Andres-Lacueva C (2018). Metabotypes of response to bariatric surgery independent of the magnitude of weight loss. PLoS One.

[22] Wijayatunga NN, Sams VG, Dawson JA, Mancini ML, Mancini GJ, Moustaid-Moussa N (2018). Roux-en-Y gastric bypass surgery alters serum metabolites and fatty acids in patients with morbid obesity. Diabetes Metab Res Rev.

[23] Yao J, Kovalik JP, Lai OF, Lee PC, Eng A, Chan WH, Tham KW, Lim E, Bee YM, Tan HC (2019). Comprehensive assessment of the effects of sleeve gastrectomy on glucose, lipid, and amino acid metabolism in Asian individuals with morbid obesity. Obes Surg.

[24] Yoshida N, Kitahama S, Yamashita T, Hirono Y, Tabata T, Saito Y, Shinohara R, Nakashima H, Emoto T, Hirota Y, Takahashi T, Ogawa W, Hirata KI (2021). Metabolic alterations in plasma after laparoscopic sleeve gastrectomy. J Diabetes Investig.

[25] Arora T, Velagapudi V, Pournaras DJ, Welbourn R, le Roux CW, Orešič M, Bäckhed F (2015). Roux-en-Y gastric bypass surgery induces early plasma metabolomic and lipidomic alterations in humans associated with diabetes remission. PLoS One.

[26] Dadson P, Rebelos E, Honka H, Juárez-Orozco LE, Kalliokoski KK, Iozzo P, Teuho J, Salminen P, Pihlajamäki J, Hannukainen JC, Nuutila P (2020). Change in abdominal, but not femoral subcutaneous fat CT-radiodensity is associated with improved metabolic profile after bariatric surgery. Nutr Metab Cardiovasc Dis.

[27] Gralka E, Luchinat C, Tenori L, Ernst B, Thurnheer M, Schultes B (2015). Metabolomic fingerprint of severe obesity is dynamically affected by bariatric surgery in a procedure-dependent manner. Am J Clin Nutr.

[28] Khoo CM, Muehlbauer MJ, Stevens RD, Pamuklar Z, Chen J, Newgard CB, Torquati A (2014). Postprandial metabolite profiles reveal differential nutrient handling after bariatric surgery compared with matched caloric restriction. Ann Surg.

[29] Kwon Y, Jang M, Lee Y, Ha J, Park S (2021). Metabolomic analysis of the improvements in insulin secretion and resistance after sleeve gastrectomy: Implications of the novel biomarkers. Obes Surg.

[30] Laferrère B, Reilly D, Arias S, Swerdlow N, Gorroochurn P, Bawa B, Bose M, Teixeira J, Stevens RD, Wenner BR, Bain JR, Muehlbauer MJ, Haqq A, Lien L, Shah SH, Svetkey LP, Newgard CB. Differential metabolic impact of gastric bypass surgery versus dietary intervention in obese diabetic subjects despite identical weight loss. Sci Transl Med. 2011;3(80):80re82. 10.1126/scitranslmed.3002043. 10.1126/scitranslmed.3002043 PMC3656497 21525399

[31] Lips MA, Van Klinken JB, van Harmelen V, Dharuri HK, t Hoen PA, Laros JF, van Ommen GJ, Janssen IM, Van Ramshorst B, Van Wagensveld BA, Swank DJ, Van Dielen F, Dane A, Harms A, Vreeken R, Hankemeier T, Smit JW, Pijl H, Willems van Dijk K. Roux-en-Y gastric bypass surgery, but not calorie restriction, reduces plasma branched-chain amino acids in obese women independent of weight loss or the presence of type 2 diabetes. Diabetes Care. 2014;37(12):3150–6. 10.2337/dc14-0195. 10.2337/dc14-0195 25315204

[32] Lopes TI, Geloneze B, Pareja JC, Calixto AR, Ferreira MM, Marsaioli AJ (2015). Blood metabolome changes before and after bariatric surgery: a (1)H NMR-based clinical investigation. OMICS.

[33] Tan HC, Khoo CM, Tan MZ, Kovalik JP, Ng AC, Eng AK, Lai OF, Ching JH, Tham KW, Pasupathy S (2016). The effects of sleeve gastrectomy and gastric bypass on branched-chain amino acid metabolism 1 year after bariatric surgery. Obes Surg.

[34] Tan HC, Hsu JW, Kovalik JP, Eng A, Chan WH, Khoo CM, Tai ES, Chacko S, Jahoor F (2020). Branched-chain amino acid oxidation is elevated in adults with morbid obesity and decreases significantly after sleeve gastrectomy. J Nutr.

[35] Cabré N, Luciano-Mateo F, Baiges-Gayà G, Fernández-Arroyo S, Rodríguez-Tomàs E, Hernández-Aguilera A, París M, Sabench F, Del Castillo D, López-Miranda J, Menéndez JA, Camps J, Joven J (2020). Plasma metabolic alterations in patients with severe obesity and non-alcoholic steatohepatitis. Aliment Pharmacol Ther.

[36] Hubal MJ, Nadler EP, Ferrante SC, Barberio MD, Suh JH, Wang J, Dohm GL, Pories WJ, Mietus-Snyder M, Freishtat RJ (2017). Circulating adipocyte-derived exosomal microRNAs associated with decreased insulin resistance after gastric bypass. Obesity.

[37] Samczuk P, Luba M, Godzien J, Mastrangelo A, Hady HR, Dadan J, Barbas C, Gorska M, Kretowski A, Ciborowski M (2018). Gear mechanism of bariatric interventions revealed by untargeted metabolomics. J Pharm Biomed Anal.

[38] Mendonça Machado N, Torrinhas RS, Sala P, Ishida RK, Guarda I, Moura EGH, Sakai P, Santo MA, Linetzky WD (2020). Type 2 diabetes metabolic improvement after Roux-en-Y gastric bypass may include a compensatory mechanism that balances fatty acid β and ω oxidation. JPEN J Parenter Enteral Nutr.

[39] Jüllig M, Yip S, Xu A, Smith G, Middleditch M, Booth M, Babor R, Beban G, Murphy R (2014). Lower fetuin-A, retinol binding protein 4 and several metabolites after gastric bypass compared to sleeve gastrectomy in patients with type 2 diabetes. PLoS One.

[40] Ocaña-Wilhelmi L, Cardona F, Garrido-Sanchez L, Fernandez-Garcia D, Tinahones FJ, Ramos-Molina B (2020). Change in serum polyamine metabolome pattern after bariatric surgery in obese patients with metabolic syndrome. Surg Obes Relat Dis.

[41] Sarosiek K, Pappan KL, Gandhi AV, Saxena S, Kang CY, McMahon H, Chipitsyna GI, Tichansky DS, Arafat HA (2016). Conserved metabolic changes in nondiabetic and type 2 diabetic bariatric surgery patients: Global metabolomic pilot study. J Diabetes Res.

[42] Oberbach A, Blüher M, Wirth H, Till H, Kovacs P, Kullnick Y, Schlichting N, Tomm JM, Rolle-Kampczyk U, Murugaiyan J, Binder H, Dietrich A, von Bergen M (2011). Combined proteomic and metabolomic profiling of serum reveals association of the complement system with obesity and identifies novel markers of body fat mass changes. J Proteome Res.

[43] Herzog K, Berggren J, Al Majdoub M, Balderas Arroyo C, Lindqvist A, Hedenbro J, Groop L, Wierup N, Spégel P (2020). Metabolic effects of gastric bypass surgery: Is it all about calories?. Diabetes.

[44] Fiamoncini J, Fernandes Barbosa C, Arnoni Junior JR, Araújo Junior JC, Taglieri C, Szego T, Gelhaus B, Possolo de Souza H, Daniel H, Martins de Lima T. Roux-en-Y gastric bypass surgery induces distinct but frequently transient effects on acylcarnitine, bile acid and phospholipid levels. Metabolites. 2018;8(4). 10.3390/metabo8040083. 10.3390/metabo8040083 PMC6316856 30477108

[45] Mika A, Kaczynski Z, Stepnowski P, Kaczor M, Proczko-Stepaniak M, Kaska L, Sledzinski T. Potential application of H-1 NMR for routine serum lipidome analysis - evaluation of effects of bariatric surgery. Sci Rep. 2017;7. 10.1038/s41598-017-15346-0. 10.1038/s41598-017-15346-0 PMC5686116 29138414

[46] Ahlin S, Cefalù C, Bondia-Pons I, Capristo E, Marini L, Gastaldelli A, Mingrone G, Nolan JJ (2019). Bile acid changes after metabolic surgery are linked to improvement in insulin sensitivity. Br J Surg.

[47] Ramos-Molina B, Castellano-Castillo D, Alcaide-Torres J, Pastor Ó, de Luna DR, Salas-Salvadó J, López-Moreno J, Fernández-García JC, Macías-González M, Cardona F, Tinahones FJ (2018). Differential effects of restrictive and malabsorptive bariatric surgery procedures on the serum lipidome in obese subjects. J Clin Lipidol.

[48] Ahmad NN, Pfalzer A, Kaplan LM (2013). Roux-en-Y gastric bypass normalizes the blunted postprandial bile acid excursion associated with obesity. Int J Obes.

[49] Kindel TL, Krause C, Helm MC, McBride CL, Oleynikov D, Thakare R, Alamoudi J, Kothari V, Alnouti Y, Kohli R (2018). Increased glycine-amidated hyocholic acid correlates to improved early weight loss after sleeve gastrectomy. Surg Endosc Other Interv Tech.

[50] Kayser BD, Lhomme M, Dao MC, Ichou F, Bouillot JL, Prifti E, Kontush A, Chevallier JM, Aron-Wisnewsky J, Dugail I, Clément K (2017). Serum lipidomics reveals early differential effects of gastric bypass compared with banding on phospholipids and sphingolipids independent of differences in weight loss. Int J Obes (Lond).

[51] Friedrich N, Budde K, Wolf T, Jungnickel A, Grotevendt A, Dressler M, Völzke H, Blüher M, Nauck M, Lohmann T, Wallaschofksi H (2012). Short-term changes of the urine metabolome after bariatric surgery. OMICS.

[52] Pereira SS, Jarak I, Carvalho RA, Oliveira PF, Alves MG, Guimarães M, Almeida R, Pereira AM, Albrechtsen NJW, Holst JJ, Nora M, Monteiro MP (2020). Different malabsorptive obesity surgery interventions result in distinct postprandial amino acid metabolomic signatures. Obes Surg.

[53] Jarak I, Pereira SS, Carvalho RA, Oliveira PF, Alves MG, Guimarães M, Albrechtsen NJW, Holst JJ, Nora M, Monteiro MP (2020). Gastric bypass with different biliopancreatic limb lengths results in similar post-absorptive metabolomics profiles. Obes Surg.

[54] Kwon Y, Jang M, Lee Y, Ha J, Park S. Amino acid metabolites and slow weight loss in the early postoperative period after sleeve gastrectomy. J Clin Med. 2020;9(8). 10.3390/jcm9082348. 10.3390/jcm9082348 PMC7463855 32717870

[55] Abidi W, Nestoridi E, Feldman H, Stefater M, Clish C, Thompson CC, Stylopoulos N (2020). Differential metabolomic signatures in patients with weight regain and sustained weight loss after gastric bypass surgery: a pilot study. Dig Dis Sci.

[56] Shantavasinkul PC, Muehlbauer MJ, Bain JR, Ilkayeva OR, Craig DM, Newgard CB, Svetkey LP, Shah SH, Torquati A (2018). Improvement in insulin resistance after gastric bypass surgery is correlated with a decline in plasma 2-hydroxybutyric acid. Surg Obes Relat Dis.

[57] Samczuk P, Hady HR, Adamska-Patruno E, Citko A, Dadan J, Barbas C, Kretowski A, Ciborowski M. In-and-out molecular changes linked to the type 2 diabetes remission after bariatric surgery: an influence of gut microbes on mitochondria metabolism. Int J Mol Sci. 2018;19(12). 10.3390/ijms19123744. 10.3390/ijms19123744 PMC6321270 30477251

[58] Lopes TI, Geloneze B, Pareja JC, Calixto AR, Ferreira MM, Marsaioli AJ (2016). Omics prospective monitoring of bariatric surgery: Roux-En-Y gastric bypass outcomes using mixed-meal tolerance test and time-resolved (1)H NMR-based metabolomics. OMICS.

[59] Yu H, Ni Y, Bao Y, Zhang P, Zhao A, Chen T, Xie G, Tu Y, Zhang L, Su M, Wei L, Jia W, Jia W (2015). Chenodeoxycholic acid as a potential prognostic marker for Roux-en-Y gastric bypass in Chinese obese patients. J Clin Endocrinol Metab.

[60] Zhao L, Ni Y, Yu H, Zhang P, Zhao A, Bao Y, Liu J, Chen T, Xie G, Panee J, Chen W, Rajani C, Wei R, Su M, Jia W, Jia W (2017). Serum stearic acid/palmitic acid ratio as a potential predictor of diabetes remission after Roux-en-Y gastric bypass in obesity. Faseb j.

[61] Li QR, Wang ZM, Albrechtsen NJW, Wang DD, Su ZD, Gao XF, Wu QQ, Zhang HP, Zhu L, Li RX, Jacobsen S, Jorgensen NB, Dirksen C, Bojsen-Moller KN, Petersen JS, Madsbad S, Clausen TR, Diderichsen B, Chen LN, Holst JJ, Zeng R, Wu JR (2018). Systems signatures reveal unique remission-path of type 2 diabetes following Roux-en-Y gastric bypass surgery. EBioMedicine.

[62] Ha J, Jang M, Kwon Y, Park YS, Park DJ, Lee JH, Lee HJ, Ha TK, Kim YJ, Han SM, Han SU, Heo Y, Park S. Metabolomic profiles predict diabetes remission after bariatric surgery. J Clin Med. 2020;9(12). 10.3390/jcm9123897. 10.3390/jcm9123897 PMC7760750 33271740

[63] Kwon HN, Lee YJ, Kang JH, Choi JH, An YJ, Kang S, Lee DH, Suh YJ, Heo Y, Park S (2014). Prediction of glycated hemoglobin levels at 3 months after metabolic surgery based on the 7-day plasma metabolic profile. PLoS One.

[64] Rauschert S, Uhl O, Koletzko B, Hellmuth C (2014). Metabolomic biomarkers for obesity in humans: a short review. Ann Nutr Metab.

[65] Newgard CB, An J, Bain JR, Muehlbauer MJ, Stevens RD, Lien LF, Haqq AM, Shah SH, Arlotto M, Slentz CA, Rochon J, Gallup D, Ilkayeva O, Wenner BR, Yancy WS, Eisenson H, Musante G, Surwit RS, Millington DS, Butler MD, Svetkey LP (2009). A branched-chain amino acid-related metabolic signature that differentiates obese and lean humans and contributes to insulin resistance. Cell Metab.

[66] She P, Van Horn C, Reid T, Hutson SM, Cooney RN, Lynch CJ (2007). Obesity-related elevations in plasma leucine are associated with alterations in enzymes involved in branched-chain amino acid metabolism. Am J Physiol Endocrinol Metab.

[67] Guasch-Ferré M, Hruby A, Toledo E, Clish CB, Martínez-González MA, Salas-Salvadó J, Hu FB (2016). Metabolomics in prediabetes and diabetes: a systematic review and meta-analysis. Diabetes Care.

[68] Felig P, Marliss E, Cahill GF (1969). Plasma amino acid levels and insulin secretion in obesity. N Engl J Med.

[69] Alves A, Bassot A, Bulteau A-L, Pirola L, Morio B (2019). Glycine metabolism and its alterations in obesity and metabolic diseases. Nutrients.

[70] Wang-Sattler R, Yu Z, Herder C, Messias AC, Floegel A, He Y, Heim K, Campillos M, Holzapfel C, Thorand B, Grallert H, Xu T, Bader E, Huth C, Mittelstrass K, Döring A, Meisinger C, Gieger C, Prehn C, Roemisch-Margl W, Carstensen M, Xie L, Yamanaka-Okumura H, Xing G, Ceglarek U, Thiery J, Giani G, Lickert H, Lin X, Li Y, Boeing H, Joost HG, de Angelis MH, Rathmann W, Suhre K, Prokisch H, Peters A, Meitinger T, Roden M, Wichmann HE, Pischon T, Adamski J, Illig T (2012). Novel biomarkers for pre-diabetes identified by metabolomics. Mol Syst Biol.

[71] Rowland I, Gibson G, Heinken A, Scott K, Swann J, Thiele I, Tuohy K (2018). Gut microbiota functions: Metabolism of nutrients and other food components. Eur J Nutr.

[72] Magne F, Gotteland M, Gauthier L, Zazueta A, Pesoa S, Navarrete P, Balamurugan R. The Firmicutes/Bacteroidetes ratio: a relevant marker of gut dysbiosis in obese patients? Nutrients. 2020;12(5). 10.3390/nu12051474. 10.3390/nu12051474 PMC7285218 32438689

[73] Smith EA, Macfarlane GT (1996). Enumeration of human colonic bacteria producing phenolic and indolic compounds: Effects of pH, carbohydrate availability and retention time on dissimilatory aromatic amino acid metabolism. J Appl Bacteriol.

[74] Zhang YJ, Li S, Gan RY, Zhou T, Xu DP, Li HB (2015). Impacts of gut bacteria on human health and diseases. Int J Mol Sci.

[75] Murphy R, Tsai P, Jüllig M, Liu A, Plank L, Booth M (2017). Differential changes in gut microbiota after gastric bypass and sleeve gastrectomy bariatric surgery vary according to diabetes remission. Obes Surg.

[76] Ohland CL, Jobin C (2015). Microbial activities and intestinal homeostasis: a delicate balance between health and disease. Cell Mol Gastroenterol Hepatol.

[77] Pereira PR, Guimarães M, Morais T, Pereira SS, Nora M, Monteiro MP (2018). Diabetic and elder patients experience superior cardiovascular benefits after gastric bypass induced weight loss. Front Endocrinol (Lausanne).

[78] Ammar W, Basset HA, Al Faramawy A, Hegazy T, Sharaf Y (2020). Bariatric surgery and cardiovascular outcome. Egypt Heart J.

[79] Trøseid M, Hov JR, Nestvold TK, Thoresen H, Berge RK, Svardal A, Lappegård KT (2016). Major increase in microbiota-dependent proatherogenic metabolite TMAO one year after bariatric surgery. Metab Syndr Relat Disord.

[80] He X, Slupsky CM (2014). Metabolic fingerprint of dimethyl sulfone (DMSO2) in microbial-mammalian co-metabolism. J Proteome Res.

[81] Patel DP, Krausz KW, Xie C, Beyoğlu D, Gonzalez FJ, Idle JR (2017). Metabolic profiling by gas chromatography-mass spectrometry of energy metabolism in high-fat diet-fed obese mice. PLoS One.

[82] Goncalves D, Barataud A, De Vadder F, Vinera J, Zitoun C, Duchampt A, Mithieux G (2015). Bile routing modification reproduces key features of gastric bypass in rat. Ann Surg.

[83] Thomas C, Gioiello A, Noriega L, Strehle A, Oury J, Rizzo G, Macchiarulo A, Yamamoto H, Mataki C, Pruzanski M, Pellicciari R, Auwerx J, Schoonjans K (2009). TGR5-mediated bile acid sensing controls glucose homeostasis. Cell Metab.

[84] Russell DW. Fifty years of advances in bile acid synthesis and metabolism. J Lipid Res. 2009;50Suppl(Suppl):S120–5. 10.1194/jlr.R800026-JLR200. 10.1194/jlr.R800026-JLR200 PMC2674696 18815433

[85] Watanabe M, Houten SM, Mataki C, Christoffolete MA, Kim BW, Sato H, Messaddeq N, Harney JW, Ezaki O, Kodama T, Schoonjans K, Bianco AC, Auwerx J (2006). Bile acids induce energy expenditure by promoting intracellular thyroid hormone activation. Nature.

[86] Nagao K, Yanagita T (2010). Medium-chain fatty acids: functional lipids for the prevention and treatment of the metabolic syndrome. Pharmacol Res.

[87] Han JR, Deng B, Sun J, Chen CG, Corkey BE, Kirkland JL, Ma J, Guo W (2007). Effects of dietary medium-chain triglyceride on weight loss and insulin sensitivity in a group of moderately overweight free-living type 2 diabetic Chinese subjects. Metabolism.

[88] Wein S, Wolffram S, Schrezenmeir J, Gasperiková D, Klimes I, Seböková E (2009). Medium-chain fatty acids ameliorate insulin resistance caused by high-fat diets in rats. Diabetes Metab Res Rev.

[89] Poisson J-PG, Cunnane SC. Long-chain fatty acid metabolism in fasting and diabetes: relation between altered desaturase activity and fatty acid composition. J Nutr Biochem. 1991;2(2):60–70. 10.1016/0955-2863(91)90030-9.

[90] Lemaitre RN, Fretts AM, Sitlani CM, Biggs ML, Mukamal K, King IB, Song X, Djoussé L, Siscovick DS, McKnight B, Sotoodehnia N, Kizer JR, Mozaffarian D (2015). Plasma phospholipid very-long-chain saturated fatty acids and incident diabetes in older adults: The cardiovascular health study. Am J Clin Nutr.

[91] Liang H, Tantiwong P, Sriwijitkamol A, Shanmugasundaram K, Mohan S, Espinoza S, Defronzo RA, Dubé JJ, Musi N (2013). Effect of a sustained reduction in plasma free fatty acid concentration on insulin signalling and inflammation in skeletal muscle from human subjects. J Physiol.

[92] Rauschert S, Uhl O, Koletzko B, Kirchberg F, Mori TA, Huang R-C, Beilin LJ, Hellmuth C, Oddy WH (2016). Lipidomics reveals associations of phospholipids with obesity and insulin resistance in young adults. J Clin Endocrinol Metab.

[93] Monzo-Beltran L, Vazquez-Tarragón A, Cerdà C, Garcia-Perez P, Iradi A, Sánchez C, Climent B, Tormos C, Vázquez-Prado A, Girbés J, Estáñ N, Blesa S, Cortés R, Chaves FJ, Sáez GT. One-year follow-up of clinical, metabolic and oxidative stress profile of morbid obese patients after laparoscopic sleeve gastrectomy. 8-oxo-dG as a clinical marker. Redox Biol. 2017;12:389–402. 10.1016/j.redox.2017.02.003. 10.1016/j.redox.2017.02.003 PMC5357674 28319890

[94] Iqbal J, Walsh MT, Hammad SM, Hussain MM. Sphingolipids and lipoproteins in health and metabolic disorders. Trends Endocrinol Metab. 2017;28(7):506–18. 10.1016/j.tem.2017.03.005. 10.1016/j.tem.2017.03.005 PMC5474131 28462811

