# Supplementary material for: Metabolomic signatures after bariatric surgery – a systematic review
Source: Rev Endocr Metab Disord. 2021 Dec 2;23(3):503–19. doi: 10.1007/s11154-021-09695-5 (PMC9156502; doi:10.1007/s11154-021-09695-5)
Supplement: Supplementary file 1 — Supplementary file1 (PDF 159 KB) [file 11154_2021_9695_MOESM1_ESM.pdf]

Reviews in Endocrine and Metabolic Disorders

Title: Metabolomic signatures after bariatric surgery – a systematic review

Authors: Matilde Vaz[1,2*], Sofia S. Pereira[1,2*], Mariana P. Monteiro[1,2]

[1] Endocrine & Metabolic Research, Unit for Multidisciplinary Research in Biomedicine (UMIB), University of Porto, Porto, Portugal.

[2] Department of Anatomy, School of Medicine and Biomedical Sciences (ICBAS), University of Porto, Porto, Portugal.

* Matilde Vaz and Sofia S. Pereira have contributed equally to this work.

Corresponding Author: Mariana P. Monteiro (mpmonteiro@icbas.up.pt)

Supplementary File 1 - Database search

PUBMED

(“bariatric surger*” OR “bariatric intervention*” OR “obesity surger*” OR “bariatric procedur*”OR "gastric bypass" OR "biliopancreatic diversion" OR "RYGB" OR "sleeve gastrectomy" OR "BPD" OR "BPD-DS" OR "duodenal switch" OR “Single anastomosis duodeno–ileal bypass” OR “SADI-S” OR “SADI” OR “SIPS”) AND (metabolomic* OR metabonomic* OR metabolite* OR “metabolome”) NOT (“bronchopulmonary dysplasia” OR infant* OR child* OR “gestational” OR pregnanc* OR animal* OR “rat” OR “rats” OR “mice” OR “mouse”)

SCOPUS

TITLE-ABS-KEY (“bariatric surger*” OR “bariatric intervention*” OR “obesity surger*” OR “bariatric procedur*”OR "gastric bypass" OR "biliopancreatic diversion" OR "RYGB" OR "sleeve gastrectomy" OR "BPD" OR "BPD-DS" OR "duodenal switch" OR “Single anastomosis duodeno–ileal bypass” OR “SADI-S” OR “SADI” OR “SIPS”) AND TITLE-ABS-KEY (metabolomic* OR metabonomic* OR metabolite* OR “metabolome”) AND NOT TITLE-ABS-KEY (“bronchopulmonary dysplasia” OR “Borderline personality disorder” OR infant* OR child* OR “gestational” OR pregnanc* OR animal* OR “rat” OR “rats” OR “mice” OR “mouse”)

WEB OF SCIENCE

#1: ((TS=(“bariatric surger*” OR “bariatric intervention*” OR “obesity surger*” OR “bariatric procedur*”OR "gastric bypass" OR "biliopancreatic diversion" OR "RYGB" OR "sleeve gastrectomy" OR "BPD" OR "BPD-DS" OR "duodenal switch" OR “Single anastomosis duodeno–ileal bypass” OR “SADI-S” OR “SADI” OR “SIPS”)) NOT (TS=("bronchopulmonary dysplasia " OR “Borderline personality disorder” OR “infants” OR “pregnancy” OR “children” OR “animal” OR
“gestational”))) AND LANGUAGE: (English) AND DOCUMENT TYPES: (Article)

Indexes = SCI-EXPANDED, SSCI, A&HCI, CPCI-S, CPCI-SSH, ESCI, CCR-EXPANDED, IC Timespan=All years

#2: ((TS=(metabolomic* OR metabonomic* OR metabolite* OR metabolome))) AND LANGUAGE: (English) AND DOCUMENT TYPES: (Article)

Indexes=SCI-EXPANDED, SSCI, A&HCI, CPCI-S, CPCI-SSH, ESCI, CCR-EXPANDED, IC Timespan=All years

#3 Final Search: #1 AND #2
