# Supplementary material for: Metabolomic signatures after bariatric surgery – a systematic review
Source: Rev Endocr Metab Disord. 2021 Dec 2;23(3):503–19. doi: 10.1007/s11154-021-09695-5 (PMC9156502; doi:10.1007/s11154-021-09695-5)
Supplement: Supplementary file 2 — Supplementary file2 (PDF 197 KB) [file 11154_2021_9695_MOESM2_ESM.pdf]

Reviews in Endocrine and Metabolic Disorders

Title: Metabolomic signatures after bariatric surgery – a systematic review

Authors: Matilde Vaz[1,2*], Sofia S. Pereira[1,2*], Mariana P. Monteiro[1,2]

[1] Endocrine & Metabolic Research, Unit for Multidisciplinary Research in Biomedicine (UMIB), University of Porto, Porto, Portugal.

[2] Department of Anatomy, School of Medicine and Biomedical Sciences (ICBAS), University of Porto, Porto, Portugal.

* Matilde Vaz and Sofia S. Pereira have contributed equally to this work.

Corresponding Author: Mariana P. Monteiro (mpmonteiro@icbas.up.pt)

Supplementary File 2- Summary of Newcastle-Ottawa Scale scores of the studies included for systematic review (cohort studies)

NEWCASTLE-OTTAWA QUALITY ASSESSMENT SCALE COHORT STUDIES									
FIRST AUTHOR AND YEAR	SELECTION				COMPARABILITY	OUTCOME			TOTAL SCORE
	1	2	3	4	1	1	2	3	(MAX 9)
Abidi W, 2020 [55]	0	1	1	1	2	1	1	1	8
Ahlin S, 2019 [46]	0	1	1	1	1	1	1	1	7
Ahmad NN, 2013 [48]	1	1	1	1	2	1	1	1	9
Arora T, 2015 [25]	0	1	1	1	2	1	1	1	8
Cabré N, 2020 [35]	1	1	1	1	2	1	1	1	9
Dadson P, 2020 [26]	1	1	1	1	1	1	1	1	8
Fiamoncini J, 2018 [44]	0	1	1	1	2	1	1	1	8
Friedrich N, 2012 [51]	1	1	1	1	2	1	1	0	8
Gralka E, 2015 [27]	1	1	1	1	2	1	1	1	9
Ha J, 2020 [62]	0	1	1	1	2	1	1	1	8
Herzog K, 2020 [43]	1	1	1	1	1	1	0	1	7
Hubal MJ, 2017 [36]	0	1	1	1	2	1	1	1	8
Jüllig M, 2014 [39]	0	1	1	1	2	1	1	1	8
Kayser BD, 2017 [50]	0	1	1	1	2	1	1	1	8
Khoo CM, 2014 [28]	0	1	1	1	2	1	1	1	8
Kindel TL, 2018 [49]	0	1	1	1	1	1	1	1	7
Kwon HN, 2014 [63]	0	1	1	1	2	1	0	1	7
Kwon Y, 2020 [54]	0	1	1	1	2	1	1	1	8
Kwon Y, 2021 [29]	0	1	1	1	1	1	0	1	6
Laferrère B, 2011 [30]	0	1	1	1	2	1	1	1	8
Li QR, 2018 [61]	0	1	1	1	2	1	1	1	8
Lips MA, 2014 [31]	1	1	1	1	2	1	0	1	8
Lopes T, 2015 [32]	0	1	1	1	2	1	1	1	8
Lopes T, 2016 [58]	0	1	1	1	1	1	1	1	7
Luo P, 2016 [17]	0	1	1	1	1	1	1	1	7
Magkos F, 2013 [18]	0	1	1	1	1	1	1	1	7
Mendonça Machado M, 2020 [38]	0	1	1	1	2	1	1	1	8
Mika A, 2017 [45]	0	1	1	1	2	1	1	1	8
Mutch DM, 2009 [19]	0	1	1	1	2	1	1	1	8
Narath SH, 2016 [20]	0	1	1	1	2	1	1	1	8
Oberbach A, 2011 [42]	1	1	1	1	2	1	1	1	9
Ocaña-Wilhelmi L, 2019 [40]	0	1	1	1	2	1	0	1	7
Palau-Rodriguez M, 2018 [21]	0	1	1	1	2	1	1	1	8
Ramos-Molina B, 2018 [47]	0	1	1	1	1	1	1	1	7
Samczuk P, 2018 [57]	0	1	1	1	2	1	1	1	8

Samczuk P, 2018 [37]	0	1	1	1	2	1	1	1	8
Sarosiek K, 2016 [41]	0	1	1	1	1	1	1	1	7
Shantavasinkul PC, 2018 [56]	0	0	1	1	1	1	1	1	6
Tan HC, 2016 [33]	0	1	1	1	2	1	1	1	8
Tan HC, 2020[34]	1	0	1	1	2	1	1	1	8
Wijayatunga NN, 2018 [22]	0	1	1	1	2	1	1	0	7
Yao J, 2019 [23]	1	1	1	1	2	1	0	1	8
Yoshida N, 2020 [24]	0	1	1	1	2	1	1	1	8
Yu H 2015 [59]	0	1	1	1	2	1	1	1	8
Zhao L, 2017 [60]	0	1	1	1	2	1	1	1	8
