# Supplementary material for: Metabolomic signatures after bariatric surgery – a systematic review
Source: Rev Endocr Metab Disord. 2021 Dec 2;23(3):503–19. doi: 10.1007/s11154-021-09695-5 (PMC9156502; doi:10.1007/s11154-021-09695-5)
Supplement: Supplementary file 3 — Supplementary file3 (PDF 131 KB) [file 11154_2021_9695_MOESM3_ESM.pdf]

Reviews in Endocrine and Metabolic Disorders

Title: Metabolomic signatures after bariatric surgery – a systematic review

Authors: Matilde Vaz[1,2*], Sofia S. Pereira[1,2*], Mariana P. Monteiro[1,2]

[1] Endocrine & Metabolic Research, Unit for Multidisciplinary Research in Biomedicine (UMIB), University of Porto, Porto, Portugal.

[2] Department of Anatomy, School of Medicine and Biomedical Sciences (ICBAS), University of Porto, Porto, Portugal.

* Matilde Vaz and Sofia S. Pereira have contributed equally to this work.

Corresponding Author: Mariana P. Monteiro (mpmonteiro@icbas.up.pt)

Supplementary File 3- Summary of Newcastle-Ottawa Scale scores of the studies included for systematic review (cross-sectional studies)

NEWCASTLE-OTTAWA SCALE ADAPTED FOR CROSS-SECTIONAL STUDIES								
FIRST AUTHOR AND YEAR	SELECTION				COMPARABILITY	OUTCOME		TOTAL SCORE
	1	2	3	4	1	1	1	(MAX 10)
Jarak I, 2020 [53]	0	1	1	2	2	2	1	9
Pereira SS, 2020 [52]	0	1	1	2	2	2	1	9
