# Supplementary material for: Metabolomic signatures after bariatric surgery – a systematic review
Source: Rev Endocr Metab Disord. 2021 Dec 2;23(3):503–19. doi: 10.1007/s11154-021-09695-5 (PMC9156502; doi:10.1007/s11154-021-09695-5)
Supplement: Supplementary file 4 — Supplementary file4 (PDF 234 KB) [file 11154_2021_9695_MOESM4_ESM.pdf]

Reviews in Endocrine and Metabolic Disorders

Title: Metabolomic signatures after bariatric surgery – a systematic review

Authors: Matilde Vaz[1,2*], Sofia S. Pereira[1,2*], Mariana P. Monteiro[1,2]

[1] Endocrine & Metabolic Research, Unit for Multidisciplinary Research in Biomedicine (UMIB), University of Porto, Porto, Portugal.

[2] Department of Anatomy, School of Medicine and Biomedical Sciences (ICBAS), University of Porto, Porto, Portugal.

* Matilde Vaz and Sofia S. Pereira have contributed equally to this work.

Corresponding Author: Mariana P. Monteiro (mpmonteiro@icbas.up.pt)

Supplementary file 4 - Summary of studies comparing the metabolomic profiles of patients after bariatric surgery

FIRST AUTHOR AND YEAR	EXPERIMENTAL DESIGN	SURGICAL PROCEDURE	SUBJECTS UNDER SURGERY	PRE-OPERATIVE BMI (kg/m^2)	AGE AT SURGERY (YEARS)	FEMALE: MALE	POST-OPERATIVE BMI (kg/m^2)	BIOLOGICAL SAMPLE	METABOLOMIC ANALYSIS	MAIN FINDINGS
Ahlin S, 2019 [46]	Prospective cohort study	RYGB and BPD	N=15	51.6 ± 9.6	44.3 ± 8.3	1:2	37.7 ± 7.4	Fasting plasma samples	Targeted UPLC-MS	After a mean of 185.3 (72.9) days: ↑ in total BA concentrations after both RYGB and BPD.
Ahmad NN, 2013 [48]	Prospective cohort study	RYGB	N=5	47.7 ± 7.4	44.8 ± 12.9	1:4	31.5 ± 5.0	Fasting and postprandial plasma samples	Targeted UPLC-MS	After 40 weeks: acceleration of the postprandial BA response, leading to an earlier rise and fall in glycine- and taurine-conjugated BAs after meal ingestion.<br>RYGB normalizes the blunted postprandial circulating BA
Arora T, 2015 [25]	Prospective cohort study	RYGB	N=16	48.9±1.3	47.4±1.9	11:5	Reduction of 31.3 ± 2.1%	Fasting plasma samples	GC-MS and UPLC-MS	After 4 days: ↓ most lipid species.<br>After 42 days: ↑ levels of decanoic and octanoic acids; ↑ most lipid species; ↓ SPMs (18:1/21:0 and 18:1/23:3)
Cabré N, 2020 [35]	Prospective cohort study	NA	N=270	Median: 46.4 (42.4-51.6)	Median: 49 (41-58)	67:23	NA	Fasting plasma samples	Targeted GC-MS and UHPLC	After 12 months: ↓ leucine and isoleucine, α-ketoglutarate, 3- hydroxybutyrate, malate, malonil-CoA, glutamate and pyruvate; ↑ Sucinyl-CoA and oxaloacetate.
Dadson P, 2020 [26]	Prospective cohort study	RYGB and SG	N=23	41.1 ± 4.2	42.8 ± 9.6	23:0	31.8 ± 13.5	Fasting serum samples	H NMR	After 6 months: ↓ ApoB/ApoA1, BCAA, AAA and GlycA; ↑ glycine; lipid parameters remained unchanged.
Fiamoncini J, 2018 [44]	Prospective cohort study	RYGB	N=26	44.1 ± 3.6	36.2 ± 7.8	18:8	35 ± 3.3	Fasting plasma and dried blood spots samples	Targeted LC-MS/MS (lipidomic)	After 90 days: transient ↑ in total ACs; sustained ↓ in PCs and ↑ SPMs and BAs.
Friedrich N, 2012 [51]	Prospective cohort study	RYGB and SG	N=50	Median: 48.3 (42.9-52.5)	Range: 22 to 63	NA	NA	Urine	H NMR	After 3 to 9 days: ↑ 3-HB, 2-HB, hippuric acid, trigonelline, and glycine.
Gralka E, 2015 [27]	Prospective cohort study	SG, proximal RYGB and distal RYGB	N= 106	46.2 ± 7.8	43.6 ± 1.0	77:33	31.7 ± 4.1(SG)<br>29.4 ± 5.9 (proximal RYGB)<br>30.7 ± 4.9 (distal RYGB)	Fasting serum	Untargeted H-NMR	After 12 months: ↑ arginine, glutamine and dimethyl sulfate; ↓ AAAs and BCAAs; normalization of pyruvate, methanol and isopropanol; temporary ↑ of citrate.<br>↑ TMAO at 12 months after SG only.

Herzog K, 2020 [43]	Prospective cohort study	RYGB	N=19	39.8 ± 3.3	43 ± 6.3	19:0	33.3 ± 3.1	Fasting and postprandial plasma	UPLC	After 1 day: ↓ medium-chain ACs and purines; ↑ short-chain AC 2:0, most aminoacids, carnitine and 3-HB; few lipids were altered.<br><br>After 6 weeks: ↓in 6 out of the 20 detected ACs and 3-HB; few lipids were altered.
Hubal MJ, 2017 [36]	Prospective cohort study	GB	N=6	51.2 ± 8.8	38.5 ± 6.8	6:0	32.6 ± 8.1	Plasma and serum samples	LC–MS	After 1 year: ↑ apargine. citrulline, glutamine, glycineand histidine; ↓ cysteinylglycine, glutamic acid, leucine/isoleucine, total BCAAs and Glu/Gln ratio.
Jüllig M, 2014 [39]	Prospective cohort study	RYGB and SG	N=15	42.1 ± 4.0 (RYGB)<br><br>42.3 ± 5.9 (SG)	41.0 ± 3.1 (RYGB)<br><br>46.8 ± 2.9 (SG)	14:1	NA	Fasting plasma samples	Untargeted GC-MS	3 days after RYGB: ↓ histidine, proline, citrate and decanoic acid.<br><br>3 days after SG: ↑ 2-HB and 3-methyl-2-oxo-pentanoic acid.
Kayser BD, 2017 [50]	Prospective cohort study	RYGB and LAGB	N=59	46.5 ± 1.0 (RYGB)<br><br>43.6 ± 0.7 (LAGB)	37.3 ± 1.9 (RYGB)<br><br>34.5 ± 1.6 (LAGB)	59:0	38.0 ± 1.2 (RYGB)<br><br>38.3 ± 1.0 (LAGB)	Fasting serum samples	Targeted LC-MS/MS (lipidomic)	After 3 months, ↓in the majority of lipids after both surgical procedures.
Khoo CM 2014 [28]	Prospective cohort study	RYGB	N=20	45.6 ± 2.4	47.9 ± 5.0	7:3	Reduction of 6.5 ± 1.0%	Fasting and postprandial plasma	Targeted tandem MS	10 to 14 days after, in fasting analysis:<br>- ↓ AA (proline, histidine, valine, phenylalanine, BCAA, AAA and total AA), alanine, and molar sum of C3 and C5 ACs; ↑ C2, long-chain (C14-C22), total AC, NEFA, ketones and 3-HB.<br><br>10 to 14 days after, in postprandial analysis:<br>- ↓ AA, molar sum of BCAA, aromatic and total AA; ↑ plasma C2, medium-chain, long-chain and total ACs
Kindel TL 2018 [49]	Prospective cohort study	SG	N=28	45.0 ± 6.8	45.4 ± 12.9	41:9	38.9 ± 6.3	Fasting and postprandial serum samples	UPLC–MS	After 12 weeks, ↑ multiple BA subtypes: fasting HCA (total, unamidated and G- sub-fractions), postprandial BAs (total and G-), CDCA (total and G-), DCA (total and G), postprandial MCA (total and G-) and postprandial HCA (total, unamidated and G-)
Kwon Y 2021 [29]	Prospective cohort study	RYGB	N=23	38.9 ± 5.2	41.8 ± 13.1	17:6	31.4 ± 5.5	Fasting serum samples	Targeted LC–MS	After 3 months: ↓KynP metabolites, BCAAs, AAAs and TDGMs.
Laferrère B, 2011 [30]	Prospective cohort study	GB	N=21	44.9 ± 8.7	43.3 ± 10.0	NA	Decrease of 4.60 ± 2.13	Fasting plasma samples	Targeted tandem MS	After a BMI decrease of 4.60 ± 2.13 kg/m^2 in BMI, individuals had a ↓ AAs, in particular BCAAs and related metabolites.

Lips MA, 2014 [31]	Prospective cohort study	RYGB and LAGB	N=27	43.1 ± 0.9 (LAGB)<br><br>44.2 ± 0.8 (RYGB and no T2D)<br><br>43.5± 1. 1 (RYGB and T2D)	46.3 ± 1.9 (LAGB)<br><br>48.6 ± 1.6 (RYGB and no T2D)<br><br>51.3 ± 1. 9 (RYGB and T2D)	27:0	39.0 ± 0.8 (LAGB)<br><br>36.63 ± 0.8 (RYGB and no T2D)<br><br>34.7 ± 0.8 (RYGB and T2D)	Fasting plasma samples	Targeted UPLC - tandem MS (AA analysis)	After 3 months: ↓ BCAAs in both procedures
Lopes TI, 2015 [32]	Prospective cohort study	RYGB	N=10	32.38 ± 2.11	Range: 25 to 65	1:1	25.48 ± 1.85	Fasting and postprandial plasma samples	H NMR and CG-MS	After 12 months: ↓ lactate, BCAAs, very low-density lipoprotein, low-density lipoprotein, N-acetyl-glycoproteins, and unsaturated lipid; ↑ PCs and HDL
Luo P, 2016 [17]	Retrospective cohort study	RYGB	N=35	30.8 ± 3.3	49.8± 9.9	19:16	24.3 ± 2.3	Fasting serum samples	Untargeted UPLC-MS	After 6 months:77 altered metabolites.<br>After 12 months: 88 altered metabolites (64 common with 6 months).<br><br>Alterations (mainly decrease) were observed in: AA and their derivates, FFAs, ACs, BAs, LPCs, PCs and SPMs.
Magkos F, 2013 [18]	Prospective cohort study	RYGB and LAGB	N=20	45.6 ± 6.7 (RYGB)<br><br>46.5 ± 8.8 (LAGB)	43 ± 7 (RYGB)<br><br>47 ± 14 (LAGB)	17:3	36.4 ± 5.0 (RYGB)<br><br>37.6 ± 7.3 (LAGB)	Plasma samples	MS/MS (AAs and ACs analysis)	Post-operative ↓ BCCAs, C3 and C5 acylcarnitine in both surgeries (22 ± 7 weeks after RYGB and 16 ± 2 weeks after LAGB)
Mendonça Machado N, 2020 [38]	Prospective cohort study	RYGB	N=28	Weight: 112.8 ± 15.6 Kg	Range: 18–60	28:0	Weight: 91.8 ± 12.2 Kg	Plasma samples	Untargeted GC-MS	After 3 months: ↓dicarboxylic acids (aminomalonate, fumaric acid, malic acid, oxalic acid); enriched metabolic pathways included arginine and proline metabolism, urea and TCA cycles, gluconeogenesis, malate-aspartate shuttle, and carnitine synthesis.
Mika A, 2017 [45]	Prospective cohort study	RYGB, OLGB and SG	N=16	41 ± 1.1	44 ± 3.1	NA	31 ± 1.1	Serum samples	H-NMR	After 6 months: ↓ all analyzed lipids - TGs, PLs (PCs, Pes and SPMs), total, free and esterified cholesterol, total and specific fatty acids. The most evident decrease was in 7-lathosterol.
Mutch DM, 2009 [19]	Prospective cohort study	RYGB	N=14	46.2 ± 1.7	45.4±3.6	14:0	35.1 ± 1.7	Serum samples	Untargeted GC-MS and LC-MS	After 6 months: ↓ BCAAs, ceramide, TGs and saturated fatty acids; ↑ specific SPMs, unsaturated fatty acids and PLs.

Narath SH, 2016 [20]	Prospective cohort study	RYGB	N=44	43.9 ± 5.4	46.8 ± 11.3	29:15	30.0 ± 4.4	Serum samples	Untargeted LC–HRMS	After 1 year: ↑TMAO indoxyl-sulfate, glycine and PC C40:7; ↓ BCAAs, choline, tyrosine, alanine and phenylalanine.<br><br>V pattern (only decrease at short time) observed for Creatine, LysoPC C16:1 and c18:2, Ornithine, PC C34:3, PC C36:5, PC C36:6, Sarcosine, Tryptophan and Uracil.<br><br>ʌ pattern (only increase at short time) observed for Acetylglycine, Arginine, Carnitine, Hydroxyisobutyric acid, Leucine Proline, Pantothenic acid, PC C38:6, Pyroglutamic acid and Threonine.
Oberbach A, 2011 [42]	Prospective cohort study	SG	N=14	54.00 ± 8.05	41.9 ± 13.29	9:5	36.31 ± 7.25	Fasting serum samples	Targeted MS/MS	After 6 months: ↑glutamine; ↓ phosphatidylcholine diacyl C42:0 and C18:2 carnitine.
Ocaña-Wilhelmi L, 2020 [40]	Prospective cohort study	SG	N=32	50.4 ± 7.2	44.6 ±7.6	25:7	38.1 ± 5.9	Fasting serum samples	Targeted UPLC-MS/MS	After 6 montths: ↑ polyamide metabolome: putrescine, acetyl-spermidine (N8-acetyl-spermidine, and N1,N8-diacetyl-spermidine), and N1,N12-diacetyl-spermine.
Palau-Rodriguez M, 2018 [21]	Prospective cohort study	SG and RYGB	N=39	50.52 ± 8.37	Range: 19 to 59	27:12	36.42 ± 6.14	Fasting serum samples	Untargeted LC-MS	After 1, 3 and 6 months: alteration in the indoles and derivates, AAs, glycerolipids, glycerophospholipids and fatty acids were observed.
Ramos-Molina B, 2018 [47]	Prospective cohort study	SG and BPD	N=37	47.9 ± 6.1 (SG)<br><br>41.8 ± 6.9 (BPD)	47.0 ± 6.7 (SG)<br><br>44.4 ± 8.2 (BPD)	22:15	36.5 ± 4.5 (SG)<br><br>39.7 ± 4.4 (BPD)	Fasting plasma samples	UPLC-MS (lipidomics)	After 6 months: BPD induced ↓ sphingolipids and PLs and ↑ BAs levels; SG induced ↑ sphingolipids and PLs, and no changes in BAs levels
Samczuk P, 2018 [37]	Prospective cohort study	SG and RYGB	N=54	50.92 ± 7.33 (SG)<br><br>45.79 ± 5.5 (RYGB)	49.3 ± 8.7 (SG)<br><br>50.1 ± 9.3 (RYGB)	29:25	37.33 ± 7.3 (SG)<br><br>32.61 ± 5.5 (RYGB)	Fasting serum samples	Untargeted GC-MS and LC-MS	6 months after both procedures: ↓ PCs, LPCs, Pes and LPEs; ↑ SPMs and choline.<br><br>After RYGB only: ↑ sulfate containing metabolites.<br><br>After SG only: ↑ p-cresol.
Sarosiek K, 2016 [41]	Prospective cohort study	SG and RYGB	N=15	48.74 ± 8.2 (SG with T2D)<br><br>43.54 ± 4.13 (SG and no T2D)<br><br>47.56 ± 6.61 (RYGB)	46.0 ± 12.84 (SG and T2D)<br><br>45.2 ± 12.24 (SG and noT2D)<br><br>44.4 ± 17.57 (RYGB)	12:3	NA	Fasting serum samples	Untargeted UPLC-MS/MS and GC-MS	After 28 days: ↑ histidine, trans-urocanate, cis-urocanate, pyroglutamylvaline, heme, glutathione, and its precursors, pentose phosphate pathway intermediates, derivate pentose sugars and 3-phosphoglycerate; ↓ ascarbate, various tocopherols and pyruvate.

Tan HC, 2016 [33]	Prospective cohort study	SG and RYGB	N=22	38.8 ± 1.3	SG: 36.3± 8 RYGB: 45.6 ± 9.1	13:9	NA	Fasting serum samples	Targeted LC/MS (Amino acids and acylcarnitine' analysis)	↓ BCCAs and total acylcarnitines after surgery.
Tan HC, 2020 [34]	Prospective cohort study	SG	N=8	Median: 38.5 (37.0–40.4	Median:29.5 (26.8–41.8)	5:3	Median: 30.5 (29.1–32.6)	Fasting plasma samples	Targeted GC-MS and LC- tandem MS	After 6 months, ↓ total BCAAs, acylcarnitines (C3 and C4) and leucine oxidation.
Wijayatunga NN, 2018 [22]	Prospective cohort study	RYGB	N=20	46.83 ± 6.21	37.25 ± 11.68	15:5	34.34 ±6.44	Fasting serum samples	Untargeted GC-MS and NMR	After 6 months: ↓ BCAAs, 2-aminobutyrate, butyrate, 2-HB, 3-HB. acetone, 2-methylglutarate, and 2-oxoisocaproate; ↑ alanine, glycine, pyruvate, taurine and fatty acids (C10:0, C13:0, C14:0, C15:0, and C18:0).
Yao J, 2019 [23]	Prospective cohort study	SG	N=11	39.1 ± 1.4	36.8 ± 9.1	8:3	31.5 ± 1.1	Plasma samples during insulin clamp and fasting	LC-MS	After 6 months: ↓ BCAAs, phenylalanine, glycine and tyrosine levels (during fasting and insulin clamp); ↓ methionine and glutamate/glutamine concentrations (during insulin clamp).
Yoshida N, 2021 [24]	Prospective cohort study	SG	N=15	40.8 ± 6.6	52.2 ± 6.5	12:3	33.5 ± 6.1	Fasting plasma samples	Untargeted MS	After 3 months, ↓ BCAAs, tryptophan, phenylalanine, tyrosine, choline, creatine, ornithine, hypoxanthine, 2-oxoisovaleric acid, 2-HB, N,N-dimethylglycine and uridine; ↑ citric acid, succinic acid, malic acid, arginine and glycine.

2-HB - 2-hydroxybutyrate; 3-HB - 3-Hydroxybutyrate; AAA - Aromatic amino acid; AA - Amino acid; AC - Acylcarnitine; BA - Bile acid; BCAA - Branched chain amino acids; BMI- Body mass index; BPD – Biliopancreatic diversion; CDCA - Chenodeoxycholic acid; DCA - Deoxycholic acid; DJB - Duodenal-jejunal bypass; FFA - Free fatty acids; G- - glycine amidated; GB - Gastric bypass; GC - Gas chromatography; Glu/Gln - glutamic acid/glutamine; H-NMR - Proton nuclear magnetic resonance; HCA - Hyocholic acid; HRMS - High resolution mass spectrometry; IR - Insuline Resistance; KynP - Kynurenine pathway; LAGB - Laparoscopic adjustable gastric band; LC- Liquid chromatography; LPC - Lysophosphatidylcholines; LPE - Lysophosphatidylethanolamines; MCA - Beta-muricholic acid; MS - Mass Spectrometry; NGT- Normal glucose tolerance; OLGB - Omega-loop gastric bypass; PC- Phosphatidylcholines; PE - Phosphatidylethanolamines; PL - Phospholipid; RYGB - Roux-en-Y Gastric Bypass; SG – Sleeve gastrectomy; SPM - Sphingomyelin; T2D – Type 2 diabetes; TCA - tricarboxylic acid; TDGM - tryptophan-derived gut microbial metabolites; TG - Triglycerides; UPLC - Ultra-Performance Liquid Chromatography
