# Supplementary material for: Metabolomic signatures after bariatric surgery – a systematic review
Source: Rev Endocr Metab Disord. 2021 Dec 2;23(3):503–19. doi: 10.1007/s11154-021-09695-5 (PMC9156502; doi:10.1007/s11154-021-09695-5)
Supplement: Supplementary file 5 — Supplementary file5 (PDF 216 KB) [file 11154_2021_9695_MOESM5_ESM.pdf]

Reviews in Endocrine and Metabolic Disorders

Title: Metabolomic signatures after bariatric surgery – a systematic review

Authors: Matilde Vaz[1,2*], Sofia S. Pereira[1,2*], Mariana P. Monteiro[1,2]

[1] Endocrine & Metabolic Research, Unit for Multidisciplinary Research in Biomedicine (UMIB), University of Porto, Porto, Portugal.

[2] Department of Anatomy, School of Medicine and Biomedical Sciences (ICBAS), University of Porto, Porto, Portugal.

* Matilde Vaz and Sofia S. Pereira have contributed equally to this work.

Corresponding Author: Mariana P. Monteiro (mpmonteiro@icbas.up.pt)

Supplementary File 5 - Summary of studies comparing the metabolomic profiles of patients with different outcomes (T2D and weight loss)

FIRST AUTHOR AND YEAR	EXPERIMENTAL DESIGN	SURGICAL PROCEDURE	SUBJECTS	PRE-OPERATIVE BMI (kg/m^2)	AGE AT SURGERY (YEARS)	FEMALE: MALE	POST-OPERATIVE BMI (kg/m^2)	BIOLOGICAL SAMPLE	METABOLOMIC ANALYSIS	OUTCOMES AND MAIN FINDINGS
Abidi W, 2020 [55]	Prospective cohort study	RYGB	Control group - patients with obesity (N=11)	47.7 ± 8.6	42.0 ± 11.1	1:1	Nadir BMI: 28.2 ± 4.8<br><br>Current BMI: 29.4 ± 4.6	Serum fasting samples	LC–MS	Outcomes: Patients with sustained weight loss at 5.0 ± 3.7 years (n=14) and patients with weight regain at 8.9 ± 3.0 (n=21)<br><br>Patients with sustained weigh loss:<br>↓ metabolites of fatty acids metabolism, including TGs, DGs and CEs; ↑ 3-HB and glycine<br><br>Patients with weigh regain<br>↓ metabolites related to serine, glycine and threonine pathway, phenylalanine metabolism, TCA cycle, and alanine and glutamate metabolism; ↑ other AAs.
			Patients with obesity undergoing RYGB (N=35)	49.9 ± 7.3	47.9 ± 10.7	20:1	Nadir BMI: 28.9 ± 2.9<br><br>Current BMI: 37.9 ± 5.7			
Ahlin S, 2019 [46]	Prospective cohort study	RYGB + BPD	Patients with obesity (N= 15), in which 13,3% with T2D (N= 2)	51.6 ± 9.6	44.3 ± 8.3	1:2	37.7 ± 7.4	Fasting plasma	Targeted UPLC-MS	Outcome: Patients with obesity and improved IR after a mean of 185.3 (72.9) days<br><br>↑ total BAs concentrations after RYGB and BPD.
Arora T, 2015 [25]	Prospective cohort study	RYGB	Patients with obesity (N= 16), in which 87.5% with T2D (N= 14)	51.0±2.3	48.1±2.3	3:4	Reduction of 30.5 ± 4.4 %	Fasting plasma	GC-MS and UPLC-MS	Outcomes: patients with (n=7) or without (n=7) T2D remission at 2 years<br><br>Patients with T2D remission:<br><br>Before surgery, ↑ metabolites from TCA cycle and pentose phosphate pathways, TG and PLs with LCFAs, and PEs.<br><br>After 4 days, ↑ aconitate, indole acetic acid and ribitol; ↓ most lipid species.<br><br>After 42 days, ↑ decanoic and octanoic acids; ↑ most lipid species.
				47.2±1.6	48.8±3.5	7:0	Reduction = 32.0 ± 2.3 %			
Ha J, 2020 [62]	Prospective cohort study	RYGB + SG	Patients with obesity[$] and T2D (N= 24)	39.6 ± 7.9	45.4 ± 10.2	17:7	28.8 ± 6.6	Fasting serum samples	Targeted LC–MS	Outcomes: patients with (n=14) or without (n=10) T2D remission at 1 year<br><br>Patients with T2D remission:<br><br>↑ baseline levels of L-DOPA and 3-HAA (sustained up to 3 months after surgery).<br><br>At 1 year, no differences in the levels of L-DOPA and 3-HAA between both outcomes.<br><br>No distinction between both surgery procedures.
				33.9 ± 4.5			28.9 ± 5.6			

Kindel T, 2018 [49]	Prospective cohort study	SG	Patients with obesity (N= 28), in which 21% with T2D	45.0 ± 6.8	45.4 ± 12.9	41:9	At 6 weeks: 40.7 ± 6.1<br>At 12 weeks: 38.9 ± 6.3	Fasting/postprandial serum samples	UPLC–MS	Outcome: Patients with significant weight loss<br>SG induced an early (6 weeks after) and persistent (12 weeks after) ↑ in multiple BAs subtypes:<br>-fasting HCA (total, unamidated and G- sub-fractions).<br>- post-prandial BAs (total and G-), CDCA (total and G-), DCA (total and G), MCA (total and G-), HCA (total, unamidated and G-).<br>↑ postprandial total and G-CDCA was significantly correlated to the 6 weeks BMI loss; ↑ post-prandial G-HCA was significantly correlated to increased weight loss at both 6 and 12 weeks.
Kwon H, 2014 [63]	Prospective cohort study	RYGB + DJB	Patients with obesity and T2D (N= 22)	30.8 ± 5.6<br>24.6 ± 3.1	39.8 ± 9.2<br>49.8 ± 6.9	1:1<br>7:3	27.2 ± 3.0<br>21.7 ± 2.2	Plasma samples	GC-MS and H-NMR	Outcomes: Patients with (n=10) or without (n=12) HbA1c improvement at 3 months<br>After 1 week, ↑ 3-HB in the improved group, whereas ↑ glucose and lipids (LDL, VLDL) in the non-improved group.<br>No distinction between both surgery procedures.
Kwon Y, 2020 [54]	Prospective cohort study	SG	Patients with obesity (N= 27), in which 74% with T2D (N=20)	38.7 ± 5.2	42.1 ± 12.9	17:10	At 3 months: 31.5 ± 5.1<br>At 6 months: 27.9 ± 4.6	Fasting serum samples	Targeted LC-MS	Outcome: Patients with significant weight loss<br>Before SG: ↓ serotonin and serotonin/5-HTrp ratio; ↑ 5-HIAA, 5-HIAA/serotonin ratio and BCAA (++isoleucine), were significantly associated with greater WL% at 3 and 6 months after SG.
Kwon Y, 2021 [29]	Prospective cohort study	SG	Patients with obesity and T2D (N=23)	38.9 ± 5.2	41.8 ± 13.1	17:6	31.4 ± 5.5	Fasting serum	Targeted LC–MS	Outcome: Patients with obesity and T2D, but improved IR at 3 months<br>↑ baseline KynP metabolites and ↓ after SG.<br>↓ BCAAs and AAAs after surgery.<br>↓ TDGMs after surgery.
Li QR, 2018 [61]	Prospective cohort study	RYGB	Patients with obesity (N=19), in which 47% with T2D (N=9)	41.5 ± 4.8 (no T2D)<br>43.1 ± 5.1 (T2D)	43 ± 13 (no T2D)<br>52 ± 9 (T2D)	NA	31.0 ± 5.1 (no T2D)<br>33.9 ± 7.5 (T2D)	Fasting and postprandial plasma	LC-MS/MS	Outcome: Patients with obesity and no T2D (N=10) and patients with obesity and T2D, but improved IR at 12 months (N=9)<br>After RYGB, ↓ fasting plasma BCAAs was associated with improved IR.
Lopes T, 2016 [58]	Prospective cohort study	RYGB	Patients with obesity and T2D (N=10)	32.38 ± 2.11	25 to 65	1:1	25.48 ± 1.85	Plasma samples	H-NMR	Outcome: Patients with T2D remission at 1 year<br>After RYGB: ↓ BCAAs.<br>After RYGB in postprandial analysis: ↓ VLDL, LDL, N-acetyl glycoproteins and unsaturated lipids; ↑ HDL and PC.

Luo P, 2016 [17]	Retrospective cohort study	RYGB	Patients with obesity[$] and T2D (N=35)	30.8 ± 3.3	49.8± 9.9 (6months after surgery)	19:16	24.3 ± 2.4<br>24.2 ± 2.3	Serum samples	Untargeted UPLC-MS	Outcomes: Patients with (n=23) or without (n=12) T2D remission at 1 year<br><br>Significant metabolic alterations related to FFA, acylcarnitines, AAs, BAs, and lipids species in both outcomes.<br><br>Patients with T2D remission:<br><br>↑ baseline levels of glycoursodeoxycholate sulfate, bilirubin and tryptophan.<br><br>6 months after RYGB: ↓ FFA 16:0, FFA 18:3, FFA 17:2 and hippuric acid.
Narath SH, 2016 [20]	Prospective cohort study	RYGB	Patients with obesity (N= 44), in which 54,5% with T2D (N= 24)	43.9 ± 5.4	46.8 ± 11.3	29:15	At 1-3 weeks: 40.8 ± 5.2 (SD)<br><br>At 1 year: 30 ± 4.4 (SD)	Serum samples	Untargeted LC–HRMS	Outcomes: Patients with high or low WL at 1 year (above or below the median of the WL); patients with (N=15) or without (N=9) T2D remission at 1 year.<br><br>After 1 year: ↓creatine, ornithine, arginine and valine in patients with high WL; ↓ sarcosine, alanine, leucyl-proline and pyroglutamic acid In patients with T2D remission.
Samczuk P, 2018 [57]	Prospective cohort study	SG	Patients with obesity and T2D (N=20)	49 ± 4.5<br>51 ± 8.3	47 ± 10.3<br>51 ± 11.4	4:7<br>4:5	37 ± 3.6<br>43 ± 7.6	Fasting serum	Untargeted GC-MS and LC-MS	Outcomes: Patients with quicker (n=11) or slower (n=9) remission of T2D<br><br>Quicker responders:<br><br>↓ baseline LPC and LPE.<br><br>Postoperative significantly changed metabolites can be classified mainly as PC, LPC, PE, LPE, AAs, organic acids, sugars and metabolites related to gutmicrobiota metabolism.
Shantavasinkul PC, 2018 [56]	Prospective cohort study	RYGB	Control group - patients with obesity in a WLMP (N=11)	38.2 ± 4.6	56.5 ± 11.4	4:7	Decrease of 7.5 ± 1.2	Plasma samples	Non-targeted GC/MS and targeted MS	Outcomes: Patients with weight loss and improved HOMA-IR after RYGB (N=11) and patients with similar weight loss after WLMP (N=11)<br><br>Patients undergoing RYGB:<br>↑ baseline 2-HB.<br>After 6 months, ↓ 2-HB, 3-HB, acetiacetic acid, CMPF, and hypoxanthine.<br>After 6 months, ↓ BCAAs; significant correlation between improved HOMA-IR and ↓ valine and BCAAs.
			Patients with obesity and T2D undergoing RYGB (n=11)	41.3 ± 3.0	50.5 ± 7.8	8:3	Decrease of 8.9 ± 1.9			

Tan HC, 2020 [34]	Prospective cohort study	SG	Control group - patients with normal weight (N=10)	Median: 23.2 (22.1–23.7)	Median: 30.3 (25.1–36.1)	0:10	NA	Fasting plasma samples	Targeted GC-MS and LC-tandem MS	Outcome: patient with weight loss and improved IR resistance at 6 months after SG (n=8)<br><br>Patients undergoing SG:<br>After 6 months, ↓ BCAAs, acylcarnitines (C3 and C4) and leucine oxidation.
			Patients with obesity and IR undergoing SG (N=8)	Median: 38.5 (37.0–40.4)	Median: 29.5 (26.8–41.8)	5:3	Median: 30.5 (29.1–32.6)			
*Yu H, 2015 [59]	Retrospective cohort study	RYGB	Patients with obesity[$] and T2D (N=38)	32.8 ± 3.9	43.7 ± 12.8	1:1	24.9 ± 2.9	Fasting serum samples	Targeted UPLC	Outcomes: Patients with (n=26) or without (n=12) T2D remission at 2 years<br><br>Patients with TD2 remission:<br>↑ baseline CDCA relative to the total BAs.<br><br>Patients with no TD2 remission:<br>1 year after RYGB, ↓ total BAs.
				31.0 ± 3.5		7:5	24.9 ± 2.5			
*Zhao L, 2017 [60]	Prospective cohort study	RYGB	Patients with obesity[$] and T2D (N=38)	32.8 ± 3.9	43.7 ± 12.8	1:1	24.4 ± 2.8	Fasting serum samples	Targeted UPLC-QTOFMS (free fatty acids)	Outcomes: Patients with (n=26) or without (n=12) T2D remission at 2 years<br><br>Patients with T2D remission:<br>↑ baseline ratios of stearic/palmitic acid (C18:0/C16:0) and eicosadienoic/linoleic acid (C20:2 n6/C18:2 n6).
				31.0 ± 3.5		7:5	24.8 ± 2.4			

* - Both studies had the same participants; $ - Asian cohort. Anthropometric characteristics of patients with good and poor outcomes are presented in green or red, respectively. Abbreviations: 2-HB - 2-hydroxybutyrate; 3-HAA - 3-hydroxyanthranilic acid; 3-HB - 3-Hydroxybutyrate; 5-HIAA - 5-hydroxyindoleacetic acid; 5-HTrp - 5-hydroxytryptophan; AAA - Aromatic amino acid; AA - Amino acid; BA - Bile acid; BCAA - Branched chain amino acids; BMI- Body mass index; BPD – Biliopancreatic diversion; CDCA - Chenodeoxycholic acid; CE - Cholesterol ester; CMPF - 3- carboxy-4-methyl-5-propyl-2-furanpropionic acid; DCA - Deoxycholic acid; DG - Diglyceride; DJB - Duodenal-jejunal bypass; FFA - Free fatty acids; G- - Glycine amidated; GC - Gas chromatography; H-NMR - Proton nuclear magnetic resonance; HCA - Hyocholic acid; HRMS - High resolution mass spectrometry; IR - Insuline Resistance; KynP - Kynurenine pathway; L-DOPA - L-dihydroxyphenylalanine; LC- Liquid chromatography; LCFA - Long-chain fatty acid; LDL - Low density lipoprotein; LPC - Lysophosphatidylcholines; LPE - Lysophosphatidylethanolamines; MCA - Beta-muricholic acid; MS - Mass Spectrometry; NGT- Normal glucose tolerance; PC- Phosphatidylcholines; PE - Phosphatidylethanolamines; PL - Phospholipid; RYGB - Roux-en-Y Gastric Bypass; SG – Sleeve gastrectomy; T2D – Type 2 Diabetes; TCA - Tricarboxylic acid; TDGM - Tryptophan-derived gut microbial metabolites; TG - Triglyceride; UPLC - Ultra-Performance Liquid Chromatography; VLDL - Very low density lipoprotein; WL - Weight loss; WLMP - Weight Loss Maintenance Program.
